# Systematic Review and Meta-Analysis of Treatments on Melasma Area Severity Index and Quality of Life

**DOI:** 10.3390/pharmaceutics17121619

**Published:** 2025-12-16

**Authors:** Milena Mariano Ribeiro, Ana Cleia Cardoso da Silva, Heloise Dalagrana, Maria Eduarda A. Galiciolli, Ana Carolina Irioda, Quelen Iane Garlet, Cláudia Sirlene Oliveira

**Affiliations:** 1 Instituto de Pesquisa Pelé Pequeno Príncipe, Curitiba 80250-060, PR, Brazil; 2 Faculdades Pequeno Príncipe, Curitiba 80230-020, PR, Brazil; 3 Curso de Farmácia, Universidade Federal do Paraná, Curitiba 81531-980, PR, Brazil; 4 Programa de Pós-graduação em Farmacologia, Universidade Federal do Paraná, Curitiba 81531-980, PR, Brazil

**Keywords:** melasma, MELASQoL, MASI, quality of life, meta-analysis

## Abstract

**Background:** Melasma is a chronic skin condition resulting from increased melanogenic activity, which induces a significant emotional impact on the patient’s quality of life. The efficacy of melasma treatments depends on individual response and on the chosen therapeutic approach, which may include topical skin-lightening agents, oral drugs, and chemical peels. **Objectives:** We aimed to evaluate the reported efficacy of treatment techniques on melasma control and patients’ quality of life through a systematic review and meta-analysis, as well as to investigate a putative relationship between melasma severity and quality of life. **Methods:** Following PRISMA guidelines, we collected data from PubMed, Scopus, and Embase databases. The eligibility criteria included studies that analyzed the quality of life through the Melasma Quality of Life (MELASQoL) scale from populations of patients suffering from melasma, scored by the Melasma Area Severity Index (MASI). **Results:** We retrieved 1296 records; those that did not meet the eligibility criteria and duplicates were excluded, resulting in 41 papers that underwent qualitative analysis (information synthesis), from which 23 papers containing 34 studies were included in the meta-analysis. The meta-analysis revealed a decrease in both MASI and MELASQoL scores following oral or topical treatment, as well as the chemical peeling procedure. Spearman correlation test showed a mild positive relationship between MASI and MELASQoL scores. **Conclusions:** This study provides evidence supporting oral and topical pharmacological treatments, as well as chemical peels, as effective interventions for melasma management. Despite high heterogeneity among studies and methodological limitations, all treatment modalities analyzed improved patients’ quality of life.

## 1. Introduction

Melasma is a chronic, acquired, and recurrent skin condition resulting from increased melanogenic activity that alters epidermal pigmentation. Melasma occurs mainly in women of childbearing age and progresses predominantly in areas exposed to ultraviolet radiation. It is a multifactorial condition, most frequently caused or exacerbated by sun exposure, pregnancy, genetic predisposition, birth control pill intake, use of topical estrogen, and hormone replacement therapy [1,2,3]. Skin hyperpigmentation can induce a significant emotional impact, as changes in facial skin may negatively affect self-esteem and self-image, potentially reducing quality of life [4,5]. The consequences of melasma onset and progression are primarily linked to physical appearance, which interferes with psychosocial, family, and professional aspects of life. Because melasma spots occur mainly on the face, they often induce feelings of shame, distress, and sadness [5,6]. Such disturbances in a patient’s life may lead to social withdrawal and impaired mental health [7]. Accordingly, a study on 350 healthy adult Brazilian women resulted in a prevalence of anxiety and depression of 39 and 25%, respectively, supporting that these prevalences among women with melasma are higher than the expected [8]. This data shows that depression and anxiety have been reported as disorders strongly correlated with facial melasma.

There are several methods to assess the classification and severity of melasma. The Melasma Area Severity Index (MASI), proposed in 1994, is one of the most widely used tools to clinically quantify facial melasma severity [9,10]. The MASI score is based on the subjective evaluation of three components: area of involvement, pigmentation, and homogeneity. Four areas of the face are evaluated in MASI estimation—frontal, right malar, left malar, and mentonian regions—corresponding to 30%, 30%, 30%, and 10% of the total facial area, respectively [9]. In contrast, the Melasma Quality of Life (MELASQoL) scale is an instrument developed to assess the quality of life of patients suffering from melasma [6,11]. MELASQoL was proposed in 2003 [11] and has since been translated, adapted, and validated into the languages and cultures of several countries, such as Brazil (MELASQoL-BP) [12], Latin American countries (MELASQoL-SP) [13], Turkey (MELASQoL-TR) [14], and India (MELASQoL-HI) [15]. The MELASQoL questionnaire focuses on the life dimensions most affected by the condition, such as social life, recreation/leisure, and emotional well-being [6,11]. Patients evaluate their perspectives regarding these aspects using a scale ranging from 1 (no disturbance) to 7 (constant disturbance). The total MELASQoL score ranges from 7 to 70, with higher scores indicating poorer health-related quality of life due to melasma [11]. Zhu and colleagues [16], through a systematic review, observed that the relationship between MELASQoL and MASI scores is not well established, and the available data remain inconsistent in the literature. Therefore, statistical analyses are necessary to determine a potential relationship between MELASQoL and MASI. Evidence supporting such a relationship would help clinicians choose better treatments for their patients, addressing both skin pigmentation and quality of life.

Treatment of melasma remains challenging due to repigmentation once therapy is discontinued. Melasma prevalence in the general population is not well established, and available data suggest that it varies according to sex, ethnicity, and skin phototype [1,17]. Overall, the prevalence of melasma is around 1% in the general population. Conversely, in high-risk populations (women, pregnant women, and individuals with skin phototypes III and IV), prevalence ranges from 20% to 50% [1,17,18,19,20,21]. The efficacy of melasma treatments depends on individual responsiveness and the chosen therapeutic approach. Clinically, topical skin-lightening agents are considered first-line therapy for mild melasma. However, moderate to severe cases typically require a combination of two or more therapeutic techniques. Therapeutic approaches can be grouped into: (I) energy-free therapies, including topical pharmacological treatment (e.g., hydroquinone, triple-combination cream, azelaic acid, tranexamic acid), platelet-rich plasma, sunscreen, oral treatments (e.g., *Polypodium leucotomos*, tranexamic acid), chemical peels, and microneedling [22,23,24]; and (II) energy-dependent therapies [25], such as intense pulsed light, Q-switched laser, picosecond laser, and fractional CO_2_ laser. Energy-free therapies generally induce fewer side effects compared with energy-dependent therapies. However, the latter typically produce a faster therapeutic response and may lead to temporary melasma clearance. On the other hand, patients undergoing energy-dependent treatment are more prone to repigmentation and/or melasma worsening [24]. Although individual variability in treatment response cannot be ruled out, there is still no consensus on which therapies effectively improve both MASI scores and quality of life as measured by a validated scale. Therefore, this study aimed to evaluate the reported efficacy of different treatment techniques on melasma-affected area and patient quality of life through a systematic review and meta-analysis, as well as to investigate a potential relationship between melasma severity and patients’ quality of life.

## 2. Results

**Figure 1:**
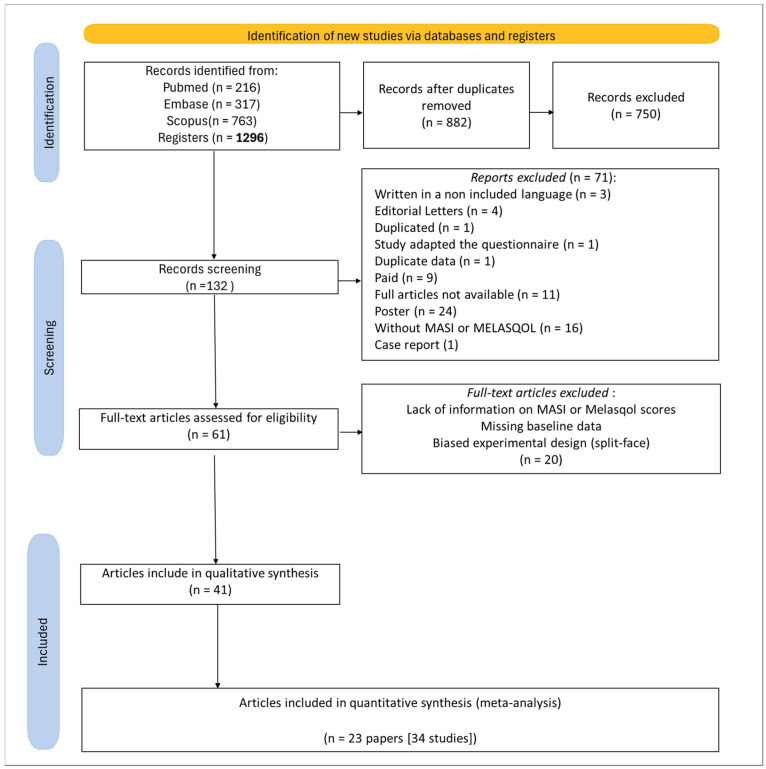
PRISMA flow diagram.

In this study, 1296 scientific records were extracted from the targeted databases (Figure 1). A total of 414 duplicates were detected and removed, resulting in 882 articles. After reading the titles and abstracts, 750 records were excluded according to the eligibility criteria. This process resulted in 132 eligible articles, which were then submitted to full-text reading. The full-text analysis led to 71 exclusions (Table S1) based on relevance to the criteria, and an additional 20 exclusions (Table S1) were made due to insufficient information regarding MASI and/or MELASQoL scores, missing baseline data, and/or biased experimental design. Therefore, we synthesized the information gathered as a qualitative analysis using the final 41 selected articles.

Among the final sample, only 10 articles evaluated the correlation between MASI and MELASQoL, and 31 articles assessed melasma before and after treatment and the putative impact on MASI and MELASQoL. Finally, 23 papers—those from which we retrieved data on the treatment effect on MASI and MELASQoL—contained 34 studies (some papers included two or more studies) evaluating a total of 1203 patients (total number at the end of the study) and were included in the quantitative analysis.

### 2.1. Quality Assessment

The risk of bias in the studies selected for the systematic review and meta-analysis is shown in Figure S1. The risk of bias analysis for non-randomized studies (ROBINS-I tool) was applied to 29 studies, in which we detected high risk of bias in 15 studies (51.7%), moderate risk in 9 studies (31.0%), and low risk in 5 studies (17.3%). This final assessment resulted from the balance between high- and low-risk domain classifications. Bias in the measurement of interventions proved to be a critical domain for the quality analysis, as 55.2% of the studies exhibited moderate to high risk of bias in this category. Conversely, in the domain of bias due to departures from intended interventions, 96.6% of all non-randomized studies demonstrated a low risk of bias.

The risk of bias in randomized studies was assessed using the RoB 2 tool (Figure S1) for the 12 randomized studies included in this systematic review. We detected high risk of bias in 4 studies (33.3%), moderate risk in 4 studies (33.3%), and low risk in 4 studies (33.3%). The high-risk studies were particularly problematic in domains 2 and 4, which assessed deviations from intended interventions and deviations in outcome measurement, respectively. In these studies, we noted a lack of information about the nature and design of the intervention, or they were open-label studies, potentially affecting the interpretation of the outcomes.

### 2.2. Sample Origin

The systematically selected studies were performed in several countries, namely, Brazil, India, South Korea, Indonesia, Peru, France, the United States of America, Mexico, Pakistan, China, South Africa, Iran, Lebanon, Germany, and Singapore. The reported sample origins were mainly from tropical and/or highly populated countries, which may have a high number of patients suffering from melasma. The major study sample evaluating the MASI and MELASQoL relationship was from China (n = 470 patients) [26] (Table 1). In the group of studies qualified for the meta-analysis, the main study sample evaluating MASI and MELASQoL before and after treatment evaluated 244 patients divided into two treatment groups [27] (Table 2).

### 2.3. Sample Characterization

Studies that only observed the MASI and MELASQoL relationship included 1343 subjects, from which 1133 (84.3%) were females, 81 (6.1%) were males, and 129 (9.6%) subjects did not disclose sex. The ages of these subjects ranged from 20 to 68 years old (Table 1). Studies evaluating MASI and MELASQoL before and after treatment included 1434 subjects ranging from 20 to 60 years old, of which 1348 (94.0%) were females, 53 (3.7%) were males, and 33 (2.3%) did not disclose sex. The ages of these subjects ranged from 20 to 60 years old (Table 2).

### 2.4. Effect of Treatment on MASI and MELASQoL

The melasma treatments analyzed for their effects on MASI and MELASQoL included interventions involving several pharmaceutical presentations (e.g., creams, serums, and plant extracts; Table 2), as well as dermatological procedures (peeling, microneedling, and intense pulsed light), or combinations of both. The pharmaceutical formulations used to treat melasma in the analyzed studies included a wide variety of substances, namely vitamin C, vitamin E, hydroquinone, fluocinolone acetonide, tretinoin, ellagic acid, hydroxyphenoxy propionic acid, yeast extract (Saccharomyces cerevisiae), salicylic acid, tranexamic acid, cysteamine, nicotinamide, arbutin, glycolic acid, kojic acid, N-methyl-2-pyrrolidone, dimethyl isosorbide, 4-n-butylresorcinol, retinol, undecyl-dimethyl-oxazoline, undecyl-enoyl-phenylalanine, thiamidol, octadecenedioic acid, and azelaic acid. The duration of melasma treatments ranged from 4 to 24 weeks. Qualitative analysis revealed that treatment with any of the aforementioned interventions resulted in moderate to high improvement in MASI and MELASQoL scores (Table 2).

**Figure 2:**
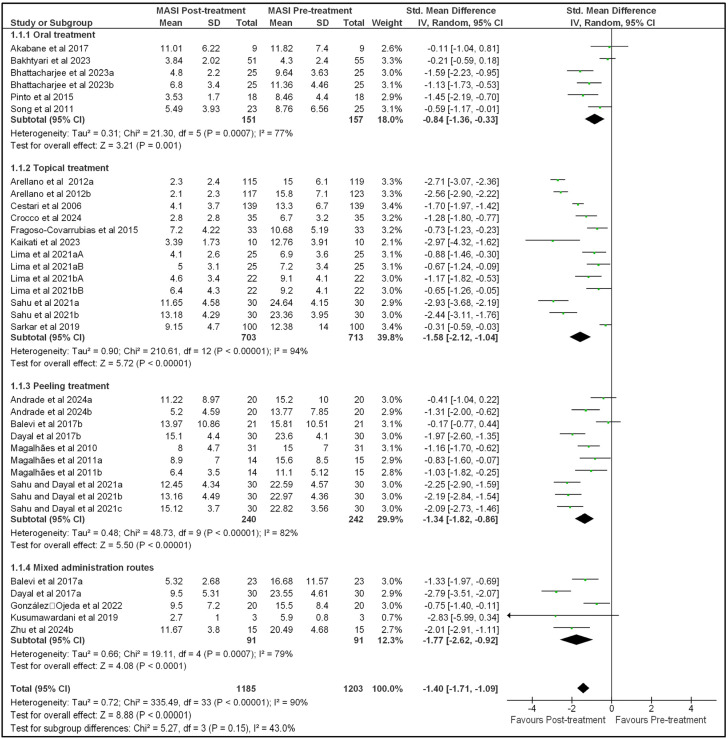
Forest plot of the standardized mean difference in Melasma Area and Severity Index (MASI) data from patients pre- and post-overall treatment and outcome adjusted for treatment route. Different studies from the same author and year were distinguished by subscripted letters (Author, YEARa or YEARb), and different studies within the same article were differentiated by an uppercase letter at the end (Author, YEARaA or YEARaB).

**Figure 3:**
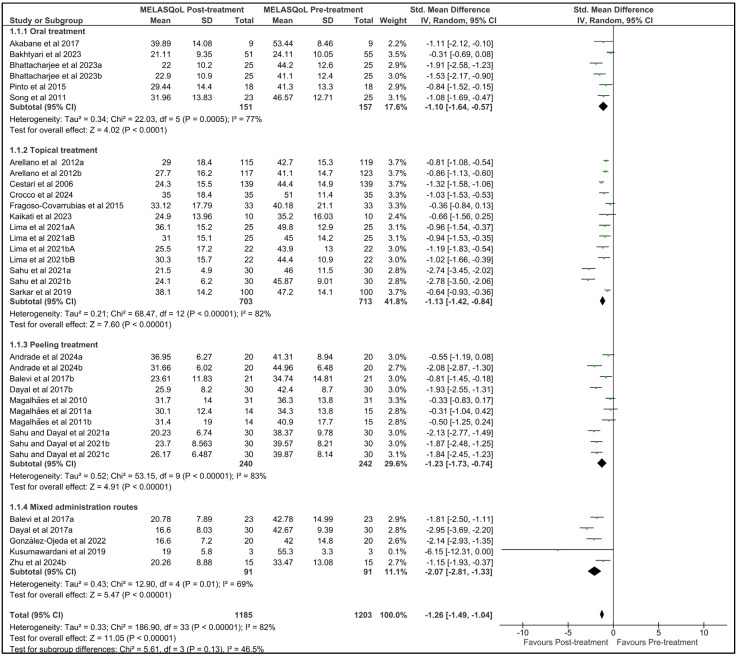
Forest plot of the standardized mean difference in Melasma Quality of Life Scale (MELASQoL) data from patients following overall treatment and outcome adjusted for treatment route. Different studies from the same author and year were distinguished by subscripted letters (Author, YEARa or YEARb), and different studies within the same article were differentiated by an uppercase letter at the end (Author, YEARaA or YEARaB).

Here, we aimed to determine whether pharmacological treatments and dermatological procedures were able to induce melasma regression and improve quality of life. For this purpose, we performed an overall meta-analysis of all 34 selected studies included in the scientific papers selected for quantitative analysis. Unfortunately, none of the studies evaluating energy-dependent therapies were included in the meta-analysis due to missing data, methodological biases, and other limitations. The forest plots from the meta-analysis on MASI and MELASQoL outcomes are shown in Figure 2 and Figure 3, respectively. Funnel plots for the meta-analysis of both MASI and MELASQoL are presented in Figure S2. Additionally, due to the high heterogeneity observed across the studies, as indicated by the I^2^ values, we identified the route of administration as a source of variability. Consequently, we categorized the treatments into four groups: oral treatments, topical treatments, peeling procedures, and mixed administration routes. These grouped studies were then subjected to a subgroup meta-analysis, controlling for each type of intervention.

The meta-analysis revealed a decrease in the MASI standardized mean difference between post-treatment and pre-treatment measures (−1.40 [−1.71, −1.09]; Z = 8.88; *p* < 0.00001; Tau^2^ = 0.72; Chi^2^ = 335.49; df = 33; I^2^ = 90%; Figure 2). More specifically, the results showed a reduction in MASI scores following oral pharmacological treatment (−0.84 [−1.36, −0.33]; Z = 3.21; *p* = 0.001; Tau^2^ = 0.31; Chi^2^ = 21.30; df = 5; I^2^ = 77%; Figure 2), topical pharmacological treatment (−1.58 [−2.12, −1.04]; Z = 5.72; *p* < 0.00001; Tau^2^ = 0.90; Chi^2^ = 210.61; df = 12; I^2^ = 94%; Figure 2), and chemical peeling procedures (−1.34 [−1.82, −0.86]; Z = 5.50; *p* < 0.00001; Tau^2^ = 0.48; Chi^2^ = 48.73; df = 9; I^2^ = 82%; Figure 2). The remaining studies included mixed administration routes (pulsed light, microneedling, used alone or combined with topical or oral treatments), with a standardized mean difference of −1.77 [−2.62, −0.92] (Z = 4.08; *p* < 0.0001; Tau^2^ = 0.66; Chi^2^ = 19.11; df = 4; I^2^ = 79%; Figure 2).

Similarly, the meta-analysis detected a decrease in the standardized mean difference of MELASQoL between post-treatment and pre-treatment measures (−1.26 [−1.49, −1.04]; Z = 11.05; *p* < 0.00001; Tau^2^ = 0.33; Chi^2^ = 186.90; df = 33; I^2^ = 82%; Figure 3). Subgroup analyses by type of intervention also showed reductions in MELASQoL scores after oral pharmacological treatment (−1.10 [−1.64, −0.57]; Z = 4.02; *p* = 0.0001; Tau^2^ = 0.34; Chi^2^ = 22.03; df = 5; I^2^ = 77%; Figure 3), topical pharmacological treatment (−1.13 [−1.42, −0.84]; Z = 7.60; *p* < 0.00001; Tau^2^ = 0.21; Chi^2^ = 68.47; df = 12; I^2^ = 82%; Figure 3), and chemical peeling procedures (−1.23 [−1.73, −0.74]; Z = 4.91; *p* < 0.00001; Tau^2^ = 0.52; Chi^2^ = 53.15; df = 9; I^2^ = 83%; Figure 3). Mixed administration route regimens yielded a standardized mean difference of −2.07 [−2.81, −1.33] (Z = 5.47; *p* < 0.00001; Tau^2^ = 0.43; Chi^2^ = 12.90; df = 4; I^2^ = 69%; Figure 3). We detected asymmetry in both MASI and MELASQoL funnel plots (Figure S2), which may indicate publication bias. This was supported by Egger’s test, which revealed significant funnel plot asymmetry (MELASQoL: z = −4.61, *p* < 0.0001; MASI: z = −3.0199, *p* = 0.0025). Additionally, heterogeneity between subgroups was not statistically significant in either MASI (I^2^ = 42.3%; *p* = 0.15; Figure 2) or MELASQoL (I^2^ = 46.5%; *p* = 0.13; Figure 3) meta-analyses, indicating that administration route alone does not fully account for the observed heterogeneity.

To further explore potential sources of heterogeneity, we performed sensitivity analyses using risk of bias and study design as grouping variables. First, we conducted meta-analyses including only low–risk-of-bias studies (Figures S3 and S4). Significant reductions remained evident, with standardized mean differences of −0.87 [−1.14, −0.59] for MASI (Z = 8.88; *p* < 0.00001; Tau^2^ = 0.15; Chi^2^ = 30.06; df = 11; I^2^ = 63%) and −1.08 [−1.43, −0.74] for MELASQoL (Z = 6.15; *p* < 0.00001; Tau^2^ = 0.27; Chi^2^ = 43.74; df = 11; I^2^ = 75%). We then analyzed only randomized studies with moderate to low risk of bias, which again showed significant reductions in MASI (−1.16 [−1.53, −0.79]; Z = 6.19; *p* < 0.00001; Tau^2^ = 0.39; Chi^2^ = 65.16; df = 13; I^2^ = 80%; Figure S5) and MELASQoL (−1.21 [−1.57, −0.85]; Z = 6.60; *p* < 0.00001; Tau^2^ = 0.37; Chi^2^ = 61.69; df = 13; I^2^ = 79%; Figure S6). Notably, -high-risk-of-bias studies showed larger effect sizes than -low-risk-of-bias studies.

Additionally, we performed a meta-analysis in which studies were grouped into short-duration treatments (2–8 weeks) and long-duration treatments (12–24 weeks). Within each time frame, we conducted subgroup analyses based on the route of administration: oral, topical, chemical peeling, and mixed administration routes. The meta-analysis by treatment duration detected a significant reduction in MASI for both short-term treatments (−1.17 [−1.60, −0.74]; Z = 5.34; *p* < 0.00001; Tau^2^ = 0.35; Chi^2^ = 41.77; df = 10; I^2^ = 76%; Figure S7) and long-term treatments (−1.48 [−2.10, −1.21]; Z = 8.89; *p* < 0.00001; Tau^2^ = 0.89; Chi^2^ = 293.13; df = 22; I^2^ = 90%; Figure S7). Among short-duration treatments, stratified analyses by route of administration indicated reductions in MASI after topical treatment (−2.15 [−3.35, −0.96]; Z = 3.52; *p* = 0.0004; Tau^2^ = 0.56; Chi^2^ = 3.28; df = 1; I^2^ = 70%; Figure S8), chemical peeling (−0.79 [−1.27, −0.32]; Z = 3.26; *p* = 0.001; Tau^2^ = 0.12; Chi^2^ = 6.21; df = 3; I^2^ = 52%; Figure S8), and mixed administration routes (−1.37 [−2.01, −0.72]; Z = 4.17; *p* < 0.0001; Tau^2^ = 0.20; Chi^2^ = 6.08; df = 3; I^2^ = 51%; Figure S8). In contrast, for long-duration treatments, stratified analyses demonstrated significant MASI reductions following oral treatment (−0.95 [−1.52, −0.39]; Z = 3.31; *p* = 0.0009; Tau^2^ = 0.32; Chi^2^ = 19.55; df = 4; I^2^ = 80%; Figure S9), topical treatment (−1.48 [−2.13, −0.83]; Z = 4.49; *p* < 0.00001; Tau^2^ = 1.12; Chi^2^ = 203.23; df = 10; I^2^ = 95%; Figure S9), chemical peeling (−1.70 [−2.29, −1.12]; Z = 5.71; *p* < 0.00001; Tau^2^ = 0.42; Chi^2^ = 24.54; df = 5; I^2^ = 80%; Figure S9), and mixed administration routes (−2.79 [−3.51, −2.07]; Z = 7.56; *p* < 0.00001; one study). Regarding MELASQoL, the meta-analysis by treatment duration similarly detected significant reductions for both short-term treatments (−1.03 [−1.41, −0.65]; Z = 5.29; *p* < 0.00001; Tau^2^ = 0.25; Chi^2^ = 33.66; df = 10; I^2^ = 70%; Figure S10) and long-term treatments (−1.36 [−1.64, −1.08]; Z = 9.47; *p* < 0.00001; Tau^2^ = 0.39; Chi^2^ = 152.98; df = 22; I^2^ = 86%; Figure S10). Among short-duration treatments, stratified analyses revealed reductions in MELASQoL following oral treatment (−1.11 [−1.12, −0.10]; Z = 2.15; *p* = 0.03; one study; Figure S11), topical treatment (−1.14 [−1.72, −0.55]; Z = 3.83; *p* = 0.0001; Tau^2^ = 0.10; Chi^2^ = 1.90; df = 1; I^2^ = 47%; Figure S11), chemical peeling (−0.48 [−0.79, −0.16]; Z = 2.97; *p* = 0.003; Tau^2^ = 0.00; Chi^2^ = 1.64; df = 3; I^2^ = 0%; Figure S11), and mixed administration routes (−1.75 [−2.37, −1.12]; Z = 5.45; *p* < 0.00001; Tau^2^ = 0.16; Chi^2^ = 5.21; df = 3; I^2^ = 42%; Figure S11). For long-duration treatments, reductions in MELASQoL were also observed after oral treatment (−1.11 [−1.71, −0.50]; Z = 3.56; *p* = 0.0004; Tau^2^ = 0.39; Chi^2^ = 21.88; df = 4; I^2^ = 82%; Figure S12), topical treatment (−1.14 [−1.48, −0.81]; Z = 6.73; *p* < 0.00001; Tau^2^ = 0.25; Chi^2^ = 60.72; df = 10; I^2^ = 84%; Figure S12), chemical peeling (−1.73 [−2.20, −1.25]; Z = 7.11; *p* < 0.00001; Tau^2^ = 0.44; Chi^2^ = 16.25; df = 5; I^2^ = 69%; Figure S12), and mixed administration routes (−2.95 [−3.69, −2.20]; Z = 7.76; *p* < 0.00001; one study).

### 2.5. MASI and MELASQoL Relationship

**Figure 4:**
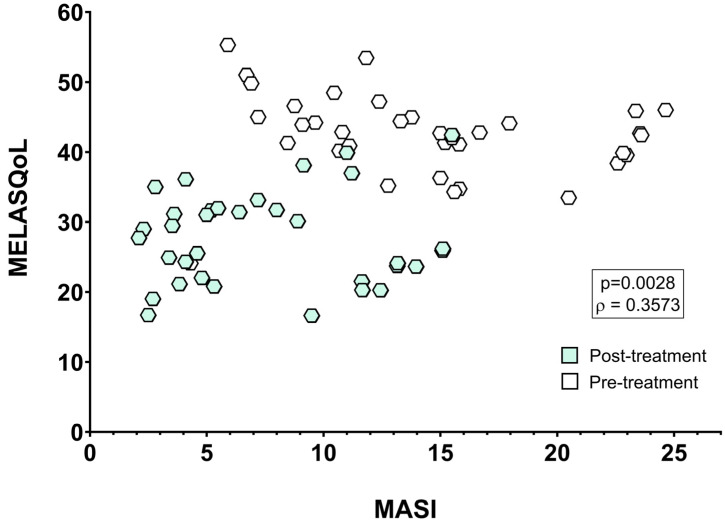
Spearman correlation between MASI and MELASQoL data from patients evaluated before and after treatments.

Studies that evaluated only the relationship between MASI and MELASQoL scores provided MASI values that ranged from 5.7 to 40.6 and MELASQoL values that ranged from 23.2 to 56.3 (Table 1). Regarding the conclusion of such analysis, five studies did not detect a significant correlation between MASI and MELASQoL scores; four studies observed a positive correlation between MASI and MELASQoL scores; and one study did not carry out a statistical test to validate the score’s relationship (Table 1). In another perspective from the collected data, we also grouped studies that evaluated the effect of any intervention for melasma control on MASI and MELASQoL scores. In this group of studies, MASI values ranged from 4.3 to 37.1 before the intervention and from 2.0 to 15.1 after a given treatment. Accordingly, MELASQoL values ranged from 24.1 to 70 before the intervention and 16.6 to 48.0 after a given treatment (Table 2). Regarding the conclusion that those studies provided, three studies did not detect a significant relationship between MASI and MELASQoL scores; two studies observed a positive correlation between MASI and MELASQoL scores; and 26 studies did not submit their data to a statistical tool to evaluate the scores’ relationship (Table 2). Due to this controversial information found in the retrieved literature, we grouped all data that met the eligibility criteria to undergo the quantitative analysis and evaluate the relationship between melasma severity and patients’ quality of life through a non-parametric correlation analysis. Therefore, we used data from the 34 studies used for the meta-analysis. Spearman correlation test showed a moderate positive relationship between MASI and MELASQoL scores from patients subjected to studies that used a variety of methodological approaches (Figure 4), showing that a high MASI score has a mild (ρ = 0.3573), nevertheless significant (*p* = 0.0028), negative impact on an individual’s quality of life (MELASQoL). To further investigate the relationship between melasma severity and quality of life, we performed a random-effects meta-regression including data from all available studies. We tested a model using delta (Δ) values to explore within-study effects and reduce baseline heterogeneity. This complementary analysis did not reveal a significant correlation between changes in MASI and MELASQoL (*p* = 0.53), although the direction of association remained consistent with the results of the non-parametric correlation analysis (ρ = 0.3573, *p* = 0.0028). Together, these findings support a mild positive relationship between melasma severity and quality of life impairment, with overall improvement following treatment despite high inter-study variability.

## 3. Discussion

Dermatologically, melasma is a common chronic and recurrent hyperpigmentation disorder—particularly prevalent among women of reproductive age—caused by hyperfunctional melanocyte activity [62]. Although benign, melasma is the subject of numerous studies due to its detrimental and psychologically distressing effects on quality of life. Women are more likely to develop the condition, seek care, and pursue treatment. Hormonal factors, particularly estrogen and progesterone fluctuations during pregnancy or the use of oral contraceptives, play a major role in melasma pathophysiology. These hormones upregulate melanogenic activity, which may partly explain the higher prevalence in women and the frequent relapse following treatment discontinuation [8]. Specialized dermatological care is often required to mitigate the discomfort associated with changes in self-perception of facial appearance and skin quality [63]. Reduced self-esteem may trigger or exacerbate mental health issues, affect interpersonal relationships, and lead to a significant decline in quality of life [64]. Indeed, melasma has been linked to psychological burden, embarrassment, and psychiatric disorders such as depression and anxiety [33].

In this context, we conducted a systematic review and meta-analysis of eligible studies, which corroborated this understanding. We found that MASI significantly correlates with quality of life, as measured by MELASQoL, and that both pharmacological treatments and dermatological procedures reported in the literature improved MASI and MELASQoL scores, despite the high heterogeneity observed across studies. All intervention types included in the eligible studies (oral and topical pharmacological treatments and chemical peeling procedures) were associated with improvement in MASI scores and patient quality of life. However, identifying a specific formulation with superior evidence proved challenging. This is largely due to the extensive variety of available formulations and the limited amount of research assessing the effects of this diverse range of interventions on MASI and MELASQoL.

It is also important to note that melasma treatment rarely achieves long-term stability when monotherapy is used [1]. The multifactorial nature of melasma—including hormonal, genetic, and inflammatory components—often requires more comprehensive and individualized therapeutic approaches. This point clearly emerged from the reviewed literature, as most patients did not respond equally to single treatments. In fact, combining different therapeutic modalities, such as topical depigmenting agents with oral antioxidants or procedural interventions, tends to yield better and more sustained outcomes. This multimodal strategy more accurately reflects real-world clinical practice, given that melasma typically requires ongoing management. However, combined protocols also make it difficult to determine which component is responsible for clinical improvement, contributing to the heterogeneity observed across studies. Even so, the overall trend indicates that combination therapies result in greater MASI reduction and higher patient satisfaction [25].

Melasma control using oral pharmacological treatments generally aims to inhibit melanin synthesis through nutrient-based and antioxidant supplementation, which reduces intracellular free radicals and suppresses melanogenesis [18,65,66]. Natural sources have also been explored in melasma management, such as *Polypodium leucotomos* [34,67], a fern native to Central and South America that has been used as an adjunctive therapy due to its antioxidant properties [67]. Oral formulations frequently include glutathione because of its strong antioxidant activity [18]. Notably, we did not identify any study in our search that evaluated oral glutathione in combination with quality-of-life outcomes using MELASQoL. Conversely, the mechanisms of action of topical treatments focus more directly on reducing melanin synthesis to decrease skin pigmentation [55]. These formulations typically contain bleaching agents and antioxidants—such as kojic acid, vitamin C, and hydroquinone—and should always be used alongside sunscreen [47], as UV radiation increases melanogenic activity [1]. Among topical strategies, photoprotection remains the first-line therapeutic approach for melasma control [18]. Recent research has also sought new depigmenting agents and pharmaceutical combinations designed to enhance efficacy while reducing adverse effects [68,69,70,71,72].

Another treatment category retrieved in this systematic review and meta-analysis was chemical peeling procedures. Chemical peels exfoliate the skin, producing controlled tissue damage followed by cytokine and inflammatory mediator release. This process increases epidermal thickness, stimulates collagen synthesis, and reorganizes structural skin components, ultimately improving skin appearance through enhanced cell renewal [73]. In the present systematic evaluation of the included studies, followed by a meta-analysis of quantitative outcomes, we found that currently available melasma treatments generally lead to decreases in melasma index and improvements in quality of life. However, when we controlled the meta-analysis by treatment type or procedure, study heterogeneity became evident, largely due to differences in therapeutic approaches and varying risk of bias. Despite these limitations, some conclusions could still be drawn regarding which treatment modalities may be more effective. Oral treatments involve a wide variety of compounds, each with distinct mechanisms of action and molecular targets, making direct comparisons across heterogeneous studies challenging. Nonetheless, we observed an overall improvement in melasma scores and quality of life among patients receiving oral medication.

Accordingly, we observed consistent benefits of topical treatments on both MASI and MELASQoL scores. Although topical interventions demonstrated a higher effect size for MASI, these findings should be interpreted with caution due to the marked heterogeneity across studies. The studies evaluating topical treatments varied considerably in experimental design, including differences in drug formulations, treatment regimens, and intervention durations. The study by Manzoni et al. [53], for instance, assessed the effects of peeling on the same patient who also underwent treatment with pulsed light using a split-face design. For this reason, the Manzoni [53] study was excluded from the meta-analysis.

Another source of bias in the topical treatment group was publication bias, as indicated by funnel plot asymmetry and Egger’s test. The Kusumawardani et al. [46] study contributed substantially to this bias and presented a wide confidence interval because it included only three patients. Nevertheless, this bias did not alter the overall treatment effect. Therefore, although the evidence supporting topical interventions is heterogeneous, the available data still support the use of topical treatments in improving melasma severity and quality of life, given their substantial effect size. It is also worth noting that both pharmacological topical agents and chemical peeling procedures are safe interventions with high toxicological reliability. In this context, we detected a significant effect of chemical peels on melasma control, supported by four high-quality studies with moderate heterogeneity. These studies provide consistent evidence demonstrating improvements in both melasma severity and patient quality of life following chemical peeling procedures.

Melasma is a dermatological condition with challenging therapeutic management and high recurrence rates, often requiring continuous treatment to prevent repigmentation [74]. Accordingly, it is essential to provide scientific evidence supporting non-invasive therapeutic options capable of positively influencing clinical outcomes while also improving quality of life. In our study, we grouped MASI results and correlated them with MELASQoL data from patients undergoing melasma treatment. We found a significant relationship indicating that patients’ quality of life improves as melasma severity decreases. Although this relationship was expected to be stronger—given the psychological burden associated with melasma—quality of life is inherently multifactorial, and melasma control may not be the only aspect influencing an individual’s well-being. Even so, both the literature and our analyses support the notion that reducing melasma severity contributes to psychological well-being. As highlighted earlier, changes in skin appearance caused by hyperpigmentation can influence self-esteem and mental health. Overall, the evidence reinforces that melasma should not be regarded merely as a cosmetic concern, but rather as a chronic condition that integrates biological, environmental, and psychological dimensions. Future research should prioritize standardizing outcome measures that incorporate both objective parameters, such as MASI, and subjective dimensions, such as quality of life, to allow for more comprehensive patient evaluation. Therefore, there is a need to create awareness around the emotional and psychological burden of melasma and educate health professionals to refer patients to psychological care if needed.

## 4. Materials and Methods

### 4.1. Systematic Review

The protocol for this systematic review, as well as its report and meta-analysis, followed the Preferred Reporting Items for Systematic Reviews and Meta-Analyses guidelines (PRISMA 2020) [75], and it was registered in the international database Prospective Registry of Systematic Reviews (PROSPERO; CRD420202214886). The complete PRISMA checklist is available in Figures S13 and S14. The acronym PICO was used to set the guiding question, where P = population (patients with melasma), I = intervention (melasma available treatments), C = comparison (with or without control group), and O = outcome (primary: melasma control efficacy; secondary: improvement in quality of life) [76].

#### 4.1.1. Search Strategy

The electronic databases chosen for this systematic review were PubMed, Scopus, and Embase using the following descriptors: “melasma” AND “quality of life”, without a filter period. The search was carried out between May and August 2024.

#### 4.1.2. Eligibility Criteria

Studies were included in the systematic review when they met the following criteria: research involving patients with melasma, the severity of which was assessed by MASI, regardless of gender or age; studies that assessed patient quality of life using the MELASQoL instrument; and articles published in English, Spanish, or Portuguese, with no time restrictions. Accordingly, exclusion criteria comprised any records that did not evaluate the quality of life of patients with melasma or did not apply the MELASQoL instrument. 

There are several tools to assess melasma severity and quality of life, such as mMASI, MSI, SHI, mexametry/colorimetry, and DLQI, SKINDEX-16. Nevertheless, we included studies applying MASI as the tool of choice to measure melasma severity and MELASQoL as the tool of choice to measure quality of life. Therefore, focusing on these two prespecified outcomes reduced heterogeneity introduced by a large variety of measurement methods and allowed a consistent synthesis of clinical trials. The pragmatic tradeoff we made was to choose the disease-specific, widely reported clinician (MASI) and patient (MELASQoL) instruments to produce clinically interpretable pooled outcomes. These two scales are the most commonly reported, disease-specific instruments in randomized and non-randomized melasma trials, maximizing comparability and feasibility of meta-analysis. Additionally, both instruments have been psychometrically evaluated in melasma populations and used in the majority of clinical studies, enabling pooled effect estimates.

#### 4.1.3. Data Extraction

The results retrieved in the electronic databases were placed in a single folder inside a Mendeley library, followed by the removal of duplicate records. The investigators (ACCS and MMR) independently examined the titles and abstracts of all records to identify potentially eligible studies. Articles deemed potentially relevant were selected. Finally, the authors (ACCS, MMR, and/or HD) independently evaluated the full-text versions of the selected articles considering the eligibility criteria. Any disagreements about the data were discussed between the two reviewers, and input from a third reviewer (CSO or QIG) was used as the casting vote. The following data were collected: title, author, year, country, study type, study period, treatment performed, treatment duration, number of participants, gender, age, MASI, and MELASQoL score. When quantitative data were not provided in the text or tables, they were extracted from the figures using the free software Graph Grabber (version 2.0.2, Quintessa Software, 2017).

### 4.2. Quality Assessment

Quality assessment was performed using the Risk Of Bias In Non-randomized Studies of Intervention (ROBINS-I) tool and Risk of Bias for Randomized Trials (Rob 2). The ROBINS-I tool has seven domains of bias, including pre-intervention, at-intervention, and post-intervention domains: bias due to confounding; bias in the selection of participants into the study; bias in classification of interventions; bias due to deviations from intended interventions; bias due to missing data; bias in the measurement of outcomes; bias in the selection of the reported result. The domains mentioned above are classified into uncertain risk, low risk, moderate risk, and high risk of bias [77]. The Rob 2 tool is structured around a fixed set of five domains, which evaluate the: randomization process; deviations from intended interventions; missing results data; outcome measurements; and selection of the reported results. Within each domain, several questions are posted to obtain information about the study characteristics relevant to the risk of bias. A proposal for judging the risk of bias arising from each domain was generated by an algorithm, based on the answers to the questions. As such, the judgment may result in a “low” or “high” risk of bias, or it may express a moderate risk of bias [78]. Furthermore, we used these quality analyses to group high-to-moderate-quality studies apart from low-quality studies to better explain high heterogeneity indexes in the meta-analysis.

### 4.3. Statistical Analysis

Meta-analysis was performed using the MASI and MELASQoL mean ± standard deviation (SD) using a 95% confidence interval as a variance measure for the analyzed studies. We analyzed the continuous variables by inverse variance method using a random-effects model and weighted means relative to the sample size of each study. Heterogeneity was assessed by the *I*-square (I^2^) index and ranked as: no heterogeneity (<25%), mild heterogeneity (25–50%), moderate heterogeneity (50–75%), and high heterogeneity (>75%) [79]. Additionally, funnel plots and Egger’s test were performed to detect asymmetry as an indication of publication bias. Subgroup analysis was performed using the administration route followed by study quality as factors for grouping the studies. Manual sensitivity analysis was performed to assess possible sources of heterogeneity. Correlations were assessed by the Spearman correlation method for non-parametric data interpreted as follows: a correlation coefficient (*ρ*) ranging from 0.1 to 0.3 indicates a weak correlation, *ρ* ranging from 0.4 to 0.6 indicates a moderate correlation and *ρ* > 0.7 indicates a strong correlation [79]. In addition to the primary correlation analysis, we performed a random-effects meta-regression to evaluate the association between MASI and MELASQoL scores across studies, considering their variability and potential baseline differences. The analysis was conducted using both absolute pre- and post-treatment scores, as well as delta values (Δ = post − pre) to account for intra-study variation. The statistical significance level was set as *p* < 0.05. All analyses were performed using the software Review Manager© version 5.4.1 (Cochrane Collaboration), R^©^ (version 2025.09.0) using the *metafor* package, and GraphPad Prism^©^ version 8.4.2.

## 5. Conclusions

In conclusion, our systematic review and meta-analysis provide evidence supporting the use of oral pharmacological treatments and topical interventions as valid therapeutic options for melasma control. Nonetheless, studies with greater methodological rigor and more robust experimental designs are still needed to generate higher-quality evidence regarding both oral and topical approaches. Additionally, we found evidence indicating that chemical peeling procedures represent a suitable alternative for melasma treatment. Although some studies presented methodological limitations, all treatment modalities analyzed in this systematic review demonstrated a positive impact on patients’ lives.

## Figures and Tables

**Table 1:** Summary of studies that evaluated a single measure of MASI and MELASQol in patients suffering from melasma.

Reference	Country	Sample ** Sex (n) Age (Years)	MASI	MELASQoL	Main Findings
Dominguez et al. [13]	USA	NI (43)	12.5 ± 6.3	47.7 ± 17.9	Data from previously treated melasma patients. Pearson’s correlation between MASI and MELASQoL showed a positive moderate correlation.
		NI (56) 41.1 ± 6.8	8.7 ± 5.7	35.7 ± 18.3	Data from patients who have never been treated for melasma. Pearson’s correlation between MASI and MELASQoL showed a positive moderate correlation.
Freitag et al. [28]	Brazil	F (84)	10.6 ± 6.6	37.5 ± 15.2	There was no correlation between MELASQoL and MASI scores.
		41.1 ± 6.8			
Harumi, Goh [29]	Singapore	F (49)	12.1 ± 6.5	25.6 ± 15.3	There was no correlation between MASI and MELASQoL.
		56.6 ± 9.1			
Jusuf et al. [7]	Indonesia	NI (30)	13.7 ± 5.0	40.0 ± 12.1	There was no correlation between the MELASQoL and MASI scores.
		39.3 ± 4.7			
Kothari et al. [30]	India	F (105)	9.1 ± 6.1	28.6 ± 13.0	There was no correlation between the MELASQoL and MASI scores.
		M (36)			
		32.3 ± 7.4			
Mpofana et al. [31]	South Africa	F (150) 47.3 ± 10.2	40.6 ± 4.9	56.3 ± 7.4	Pearson correlation matrix was used to establish the presence of multicollinearity between predictor variables. The analysis concluded a positive relationship between MASI and MELASQoL.
Qayyum et al. [32]	Pakistan	F (80) 32.4 ± 7.5	15.8 ± 8.9	46.5 ± 16.9	Spearman correlation showed a significantly strong positive correlation between MASI and MELASQoL.
Yalamanchili et al. [33]	India	F (95) 32.4 ± 7.0	5.7	28.3	The authors did not carry out a correlation test.
		M (45)			
		20.0–68.0			
Zhu et al. [26]	China	F (470) 39.7 ± 6.1	13.3 ± 7.2	23.2 ± 9.9	There was no correlation between the MELASQoL and MASI scores.

** Sample characteristics: Sex: male (M)/female (F)/not informed (NI); Age (years, mean ± SD or age range).

**Table 2:** Summary of the studies that evaluated MASI and MELASQoL at pre-treatment and post-treatment in patients suffering from melasma.

Reference/Country/Treatment	Sample ** Sex (n) Age (Years)	MASI			MELASQoL			Main Findings
		Pre	Post		Pre		Post	
Akabane et al. [34]	F (9) 37.2 ± 6.8	11.8 ± 7.4	11.0 ± 6.2		53.4 ± 8.5		38.9 ± 14.1	The authors did not carry out a correlation test.
Brazil								
Polypodium orally, twice a day for 6 weeks.								
Andrade et al. [35]	F (60)	A: 15.2 ± 10.0	A: 11.2 ± 8.9		A: 41.3 ± 8.9		A: 36.9 ± 6.3	The authors did not carry out a correlation test.
Brazil	34.2 ± 7.8	B: 13. ± 7.8	B: 5.2 ± 4.6		B: 44.9 ± 6.5		B: 31.7 ± 6.0	
A: 1% retinoic acid peeling in a conventional vehicle; B: 1% retinoic acid peeling in microemulsion; Four peeling sessions fortnightly on days 0, 15, 30, 45, and 60.								
Arellano et al. [27] Brazil and Mexico	F (231) M (11) 41.2	A: 15.0 ± 6.1 B: 15.8 ± 7.1	A: 2.3 ± 2.4 B: 2.1 ± 2.3		A: 42.7 ± 15.3 B: 41.1 ± 14.7		A: 29.0 ± 18.4 A: 27.7 ± 16.2	The authors did not carry out a correlation test.
A: Cream containing hydroquinone, fluocinolone acetonide, and tretinoin once a day for 8 weeks. Plus a maintenance phase of the same cream twice weekly for 24 weeks. B: Cream containing hydroquinone, fluocinolone acetonide, and tretinoin once a day for 8 weeks. Plus a maintenance phase of the same cream in a tapering regimen (3 times a week–1st month, 2 times a week –2nd month, 1 time a week–4 to 6th month).								
Ayres et al. [36]	NI (33)	15.5	8.8		45.4		34.6	The authors did not carry out a correlation test.
Brazil	43.0							
Cream containing ellagic acid, hydroxyphenoxy propionic acid, yeast extract, and salicylic acid twice a day for 12 weeks.								
Balevi et al. [37]	F (50)	A: 16.7 ± 11.5	A: 5.3 ± 2.6		A: 42.8 ± 15.1		A: 20.8 ± 7.8	The authors did not carry out a correlation test.
Peru	36.3 ± 10.2	B: 15.8 ± 10.5	B: 13.9 ± 10.8		B: 34.7 ± 14.8		B: 23.6 ± 11.8	
A: Salicylic acid peel and intra-lesional application of vitamin C every 2 weeks for 8 weeks. B: Salicylic acid peel every 2 weeks for 8 weeks.								
Bakhtyari et al. [38]	F (55)	4.3 ± 2.4	3.8 ± 2.0		24.1 ± 10.0		21.1 ± 9.3	The authors did not carry out a correlation test.
Iran	20.0–40.0							
Polyherbal syrup: 10 mL thrice daily before meals for 12 weeks.								
Bhattacharjee et al. [39] India	A: F (22) M (3) 36.8 ± 5.7 B: F (24) M (1) 37.6 ± 7.2	A: 9.6 ± 3.6 B: 11.4 ± 4.5	A: 4.8 ± 2.2 B: 6.8 ± 3.4		A: 44.2 ± 12.6 B: 41.1 ± 12.4		A: 22 ± 10.2 B: 22.9 ± 10.9	The authors did not carry out a correlation test.
A: 250 mg oral tranexamic acid twice daily; B: 500 mg oral tranexamic acid twice daily, followed by 12 weeks of follow-up.								
Cestari et al. [12] Brazil	F (135) M (4) 42.5 ± 7.5	13.3 ± 6.7	4.1 ± 3.7		44.4 ± 14.9		24.3 ± 15.5	Pearson’s correlation between MASI and MELASQOL was positive correlated.
Cream containing hydroquinone, fluocinolone acetonide, and tretinoin once a day for 8 weeks.								
Crocco et al. [40] Brazil	F (35) 38.5 ± 11.5	6.7 ± 3.2	2.8 ± 2.8		51.0 ± 11.4		35 ± 18.4	The authors did not carry out a correlation test.
Cream containing cysteamine and nicotinamide: Progressive application (60 min in the first month, 120 min in the second month, 180 min in the third month) for 90 days								
Dayal et al. [41] India	F (56)	A: 23.5 ± 4.6 B: 23.6 ± 4.1	A: 9.5 ± 5.3 B: 15.1 ± 4.4		A: 42.7 ± 9.3 * B: 42.4 ± 8.7 *		A: 16.6 ± 8.0 * B: 25.9 ± 8.2 *	The authors did not carry out a correlation test.
	M (4)							
	31.7 ± 5.9							
A: Trichloroacetic acid peel every 2 weeks. Plus, a cream containing vitamin C once a day for 12 weeks. B: Trichloroacetic acid peel every 2 weeks for 12 weeks.								
Fragoso-Covarrubias et al. [42] Mexico	F (33) 45.4 ± 7.2	10.7 ± 5.2	7.2 ± 4.2		40.2 ± 21.1		33.1 ± 17.8	The authors did not carry out a correlation test.
Cream containing arbutin, glycolic acid, and kojic acid once a day for 12 weeks.								
González-Ojeda et al. [43] Mexico	F (20) 41.0 ± 7.0	15.5 ± 8.4	9.5 ± 7.2		42.0 ± 14.8		16.6 ± 7.2	The authors did not carry out a correlation test.
Platelet-Rich Plasma: Three sessions every 15-day intervals.								
Hwang et al. [44] South Korea	F (39) 26.0–52.0	15.6	12.0		39.8		36.2	The authors did not carry out a correlation test.
Serum containing vitamin C, N-methyl-2-pyrrolidone, and dimethyl isosorbide twice daily for 16 weeks.								
Kaikati et al. [45] Lebanon	F (10) 45.9 ± 11.3	12.8 ± 3.9	3.4 ± 1.7		35.2 ± 16.0		24.9 ± 13.9	The authors did not carry out a correlation test.
Cream containing tranexamic acid and vitamin C for 8 weeks.								
Kusumawardani et al. [46] Indonesia	F (3) 40.0–45.0	5.9 ± 0.8	2.7 ± 1.0		55.3 ± 3.3		19.0 ± 5.8	The authors did not carry out a correlation test.
Serum containing azelaic acid, 4-n butyl resorcinol, and retinol combined with a microneedling session.								
Levy et al. 2006 [47] France	F (19) 42.7	20.3	10.2		50.8 *		42.3 *	The authors did not carry out a correlation test. * values were divided by 10
Cream containing undecyl-dimethyl-oxazoline, undecyl-enoyl-phenylalanine, vitamin C, and vitamin E, twice a day for 16 weeks.								
Lima et al. [48] Brazil	F (6) 34.0–46.0	37.1 ± 8.2	11.0 ± 2.9		70.0		32.0	The authors did not carry out a correlation test.
Microneedling sessions every 30 days. Plus, a cream containing hydroquinone, fluocinolone acetonide, and tretinoin once a day for 4 weeks.								
Lima et al. [49] Brazil	F (40) 43.0 ± 6.0	A: 9.0 (6.0–12.0) B: 6.0 (3.0–8.0)	A: 5.0 (4.0–8.0) B: 2.0 (1.0–3.0)		A: 55.0 (45.0–60.0) B: 45.0 (37.0–51.0)		A: 48.0 (26.0–53.0) B: 29.0 (16.0–45.0)	The authors did not carry out a correlation test.
A: Cream containing cysteamine, nightly application for 15 min to 2 h + 120 days with sun protection factor 50 sunscreen. with sun protection factor 50 sunscreen; B: Cream containing 4% hydroquinone, nightly application + 120 days with sun protection factor 50 sunscreen.								
Lima et al. [50] Brazil	F (50) 43.0 ± 6.0	A: 6.9 ± 3.6 B: 7.2 ± 3.4	A: 4.1 ± 2.6 B: 5.0 ± 3.1		A: 49.8 ± 12.9 B: 45.0 ± 14.2		A: 36.1 ± 15.2 B: 31.0 ± 15.1	The authors did not carry out a correlation test.
A: Cream containing thiamidol, double layer application twice a day + SPF 60 sunscreen every 3 h for 90 days; B: Cream containing 4% hydroquinone, once at bedtime + SPF 60 sunscreen every 3 h for 90 days.								
Lima et al. [51] Brazil	F (44) 39.0 ± 7.0	A: 9.1 ± 4.1 B: 9.2 ± 4.1	A: 4.6 ± 3.4 B: 6.4 ± 4.3		A: 43.9 ± 13.0 B: 44.4 ± 10.9		A: 25.5 ± 17.2 B: 30.3 ± 15.7	The authors did not carry out a correlation test.
A: 75 mg Pycnogenol, orally, twice a day. Plus a cream, daily, containing hydroquinone, tretinoin, and fluocinolone for 15 weeks. B: Cream, daily, containing hydroquinone, tretinoin, and fluocinolone for 15 weeks.								
Magalhães et al. [4] Brazil	F (31) 30.0–59.0	15.0 ± 7.0	8.0 ± 4.7		36.3 ± 13.8		31.7 ± 14.0	There was no correlation between MASI and MELASQOL.
Lactic acid chemical peel every 2 weeks for 8 weeks.								
Magalhães et al. [52] Brazil	F (27) M (3)	A: 15.6 ± 8.5 B: 11.1 ± 5.1	A: 8.9 ± 7.0 B: 6.4 ± 3.5		A: 34.3 ± 13.8 B: 40.9 ± 17.7		A: 30.1 ± 12.4 A: 31.4 ± 19.0	The authors did not carry out a correlation test.
A: 5% retinoic acid chemical peel every 2 weeks for 6 weeks; B: 10% retinoic acid chemical peel every 2 weeks for 6 weeks.								
Manzoni et al. [53] Brazil	F (14) 41.0 ± 4.6	8.4 ± 4.0	5.5 ± 3.4		42.2 ± 14.9		33.7 ± 11.6	There was no correlation between MELASQOL and MASI scores.
A: Intense pulsed light with pulse-in-pulse mode every 2 weeks for 8 weeks in the left hemiface. B: Retinoic acid peel every 2 weeks during 8 weeks in the right hemiface. Obs.: The treatments were applied to the same individual. The final values were the mean of A and B values.								
Martinez-Rico et al. [54] Mexico	A: F(22) 40.4 ± 3.9 B: F(22) 42.8 ± 4.9	A:10.4 B:10.8	A: 3.6 B: 4.2		A: 48.9 B: 42.9		A: 31.1 B: 30.6	The authors did not carry out a correlation test.
A: 325 mg oral tranexamic acid every 12 h + fluocinolone-based triple combination cream every 24 h; B: 325 mg oral tranexamic acid every 12 h for 8 weeks.								
Pinto et al. [55] Brazil	F (18) 42.0 ± 7.6	8.5 ± 4.4	3.5 ± 1.7		41.3 ± 13.3		29.4 ± 14.4	There was no correlation between MELASQOL and MASI scores.
Pycnogenol, orally, once a day for 12 weeks.								
Sahu and Dayal [56] India	F (74) M (6) A: 31.6 ± 5.6 B: 30.9 ± 5.9 C: 31.6 ± 6.3	A: 22.6 ± 4.6 B: 22.9 ± 4.4 C: 22.8 ± 3.6	A: 12.4 ± 4.3 B: 13.2 ± 4.5 C: 15.1 ± 3.7		A: 38.4 ± 9.8 B: 39.6 ± 8.2 C: 39.9 ± 8.1		A: 20.2 ± 6.7 B: 26.2 ± 6.5 C: 23.7 ± 8.6	The authors did not carry out a correlation test.
A: 30% glycolic acid chemical peel every 2 weeks for 12 weeks. B: 92% lactic acid chemical peel every 2 weeks for 12 weeks. C: 15% trichloroacetic acid every 2 weeks for 12 weeks.								
Sahu et al. [57] India	F (60) A: 34 ± 5.75 B: 32.4 ±6.2	A: 24.6 ± 4.1 B: 23.4 ± 3.9	A: 11.6 ± 4.6 B: 13.2 ± 4.3		A: 46.0 ± 11.5 B: 45.9 ± 9.0		A: 21.5 ± 4.9 B: 24.1 ± 6.2	The authors did not carry out a correlation test.
A: 30% glycolic acid chemical peel every 2 weeks. Plus, a cream containing tranexamic acid (twice a day) for 12 weeks. B: 30% glycolic acid chemical peel every 2 weeks for 12 weeks.								
Sarkar et al. [58] India	F (80) M (20) 33.3 ± 6.7	12.4 ± 14.7		9.1 ± 4.7		47.2 ± 14.0	38.1 ± 14.2	There was a positive correlation between MELASQOL and MASI scores
Sunscreen with sun protection factor 19 and PA+++, thrice a day, for 12 weeks.								
Scherdin et al. [59] Germany	F (19) M (1) 41.9 ± 11.9	8.4	4.8		28.3		19.4	The authors did not carry out a correlation test.
Cream contains octadecene dioic acid, tocopherol, and sun protection factor 30 once a day for 8 weeks.								
Song et al. [60] South Korea	F (25) 20.0–60.0	8.8 ± 6.6	5.5 ± 3.9		46.6 ± 12.7		32.0 ± 13.8	The authors did not carry out a correlation test.
Korean red ginseng powder, orally, for 24 weeks.								
Zhu et al. [61] China	F (15) 40.9 ± 1.4	20 ± 4.7	11.7 ± 3.8		33.5 ± 13.1		20.3 ± 8.9	The authors did not carry out a correlation test.
Oral tranexamic acid plus a cream containing hydroquinone for 3 weeks of treatment.								

** Sample characteristics: Sex: male (M)/female (F)/not informed (NI). * values were divided by 10.

## Data Availability

The original contributions presented in this study are included in the article/Supplementary Material. Further inquiries can be directed to the corresponding authors.

## Supplementary Materials

The following supporting information can be downloaded at: https://www.mdpi.com/article/10.3390/pharmaceutics17121619/s1, Table S1: Full-text analyzed articles/reports and reasons for exclusion.; Figure S1: Quality analysis through risk of bias on non-randomized studies and randomized studies; Figure S2: Funnel plot, respectively, from MASI and MELASQoL studies included in the meta-analysis; Figure S3: A sensitivity analysis was conducted to assess heterogeneity resulting from study bias. The MASI outcome from studies with a low risk of bias is presented in the forest and funnel plots; Figure S4: A sensitivity analysis was conducted to assess heterogeneity resulting from study bias. The MELASQoL outcome from studies with a low risk of bias is presented in the forest and funnel plots; Figure S5: A sensitivity analysis was conducted to assess heterogeneity resulting from study design and study bias (moderate and low). The MASI outcome from randomized clinical trials with a moderate to low risk of bias is presented in the forest and funnel plots; Figure S6: A sensitivity analysis was conducted to assess heterogeneity resulting from study design and study bias (moderate and low). The MELASQoL outcome from randomized clinical trials with a moderate to low risk of bias is presented in the forest and funnel plots; Figure S7: A sensitivity analysis was conducted to assess heterogeneity resulting from time of exposure. The MASI outcome from studies with short-term and long-term treatment is presented in the forest and funnel plots; Figure S8: A sensitivity analysis was conducted to assess heterogeneity resulting from short-term exposure (2 to 8 weeks) and type of exposure. The MASI outcome from studies with short-term treatment is presented in the forest and funnel plots; Figure S9: A sensitivity analysis was conducted to assess heterogeneity resulting from long-term exposure (12 to 24 weeks) and type of exposure. The MASI outcome from studies with long-term treatment is presented in the forest and funnel plots; Figure S10: A sensitivity analysis was conducted to assess heterogeneity resulting from time of exposure. The MELASQoL outcome from studies with short-term and long-term treatment is presented in the forest and funnel plots; Figure S11: A sensitivity analysis was conducted to assess heterogeneity resulting from short-term exposure (2 to 8 weeks) and type of exposure. The MELASQoL outcome from studies with short-term treatment is presented in the forest and funnel plots; Figure S12: A sensitivity analysis was conducted to assess heterogeneity resulting from long-term exposure (12 to 24 weeks) and type of exposure. The MELASQoL outcome from studies with long-term treatment is presented in the forest and funnel plots; Figure S13: PRISMA 2020 for abstracts checklist; Figure S14: PRISMA 2020 checklist.
