# Supplementary material for: Systematic Review and Meta-Analysis of Treatments on Melasma Area Severity Index and Quality of Life
Source: Pharmaceutics. 2025 Dec 16;17(12):1619. doi: 10.3390/pharmaceutics17121619 (PMC12737163; doi:10.3390/pharmaceutics17121619)
Supplement: Supplementary file 1 [file pharmaceutics-17-01619-s001.zip › pharmaceutics-3933642-supplementary.pdf]

pharmaceutics

Systematic review

Systematic Review and Meta-Analysis of Treatments on Melasma Area Severity Index and Quality of Life

Milena Mariano Ribeiro [1,2], Ana Cleia Cardoso da Silva [1,2], Heloise Dalagrana [3], Maria Eduarda A. Galiciolli [1,2], Ana Carolina Irioda [1], Quelen Iane Garlet [4,*] and Cláudia Sirlene Oliveira [1,2,*]

Table S1. Full-text analyzed articles/reports and reasons for exclusion.

Reports/articles	Reason for exclusion
Abdalla, M. A. (2021). Melasma clinical features, diagnosis, epidemiology and etiology: An update review. Siriraj Medical Journal, 73(12), 841–850. https://doi.org/10.33192/Smj.2021.109	Full article not available.
Ahmed, A., Naseer, M., Mirza, R., Akram, H., Moughal, R., & Devi, V. (2023). Frequency of factors leading to the development of melasma in female patients at tertiary care hospital. Journal of Population Therapeutics and Clinical Pharmacology, 30(18), 622–630. https://doi.org/10.53555/jptcp.v30i18.3141	Without MASI or MELASQoL
Ajjoun, M., Kharchoufa, L., Alami Merrouni, I., & Elachouri, M. (2022). Moroccan medicinal plants traditionally used for the treatment of skin diseases: From ethnobotany to clinical trials. Journal of Ethnopharmacology, 297, 115532. https://doi.org/10.1016/j.jep.2022.115532	Full article not available.
Aksoy, B., Tatlıparmak, A., Koç, E., Rastgar Shıshehgarkhaneh, L., & Işık, M. (2017). Melasma or acne: Which one affects life quality of adult patients the more? Gülhane Medical Journal, 59(3), 57-59. https://doi.org/10.26657/Gulhane.00001	Without MASI or MELASQoL
Ali, F. R., & Al-Niaimi, F. (2020). Refining the management of hyperpigmentary disorders. Clinical and Experimental Dermatology, 45(8), 1063. https://doi.org/10.1111/ced.14257	Full article not available.
Ali, R., Aman, S., Nadeem, M., & Kazmi, A. H. (2013). Quality of life in patients of melasma. Journal of Pakistan Association of Dermatologists, 23(2), 143–148.	Lack of information on MASI or MELASQoL scores.
Amatya, B., & Pokhrel, D. B. (2019). Assessment and comparison of quality of life in patients with melasma and vitiligo. Kathmandu University Medical Journal, 17(66), 114–118.	Full article not available.
Amatya, B., Jha, A. K., & Shrestha, S. (2020). Frequency of different types of facial melanoses referring to the Department of Dermatology and Venereology, Nepal Medical College and	Without MASI or MELASQoL

Teaching Hospital in 2019, and assessment of their effect on health-related quality of life. BMC Dermatology, 20(1), 4. https://doi.org/10.1186/s12895-020-00100-3	
Anderson, L., & Rodrigues, M. (2019). Quality of life in a cohort of melasma patients in Australia. The Australasian journal of dermatology, 60(2), 160–162. https://doi.org/10.1111/ajd.12969	Editorial Letter
Andra, C., Suwalska, A., Dumitrescu, A. M., Kerob, D., Delva, C., Hasse-Cieślińska, M., Solymosi, A., & Arenbergerova, M. (2020). A corrective cosmetic improves the quality of life and skin quality of subjects with facial blemishes caused by skin disorders. Clinical, Cosmetic and Investigational Dermatology, 13, 253–257. https://doi.org/10.2147/CCID.S240437	Without MASI or MELASQoL
Ayres, E., Costa, A., & Eberlin, S. (2015). A single-center, prospective, randomized trial to assess the efficacy of a skin lightening formulation containing ellagic acid, hydroxyphenoxy propionic acid, yeast extract, and salicylic acid in Brazilian subjects with melasma. Journal of the American Academy of Dermatology, 72(5, Suppl. 1), AB217. https://doi.org/10.1016/j.jaad.2015.02.882	Poster.
Ayres, E., Presti, C., Lopes, L., Fernandes, É., Valpaços, C., & Svoboda, D. (2023). Randomized study to assess the efficacy of a facial cosmetic product with nanoencapsulated cysteamine in women presenting melasma. Journal of the American Academy of Dermatology, 89(3, Suppl.), AB215. https://doi.org/10.1016/j.jaad.2023.07.861	Poster.
Bala, H. R., Nguyen, J., Ross, A., Wong, C., Paul, E., & Rodrigues, M. (2022). Randomised, Placebo-Controlled, Double-Blind Study of Oral Tranexamic Acid in the Treatment of Moderate-to-Severe Melasma in an Australian Cohort. Indian Journal of Dermatology, 67(4), 454–458. https://doi.org/10.4103/ijd.ijd_120_21	Editorial Letter
Balkrishnan, R., Kelly, A. P., McMichael, A., & Torok, H. (2004). Improved quality of life with effective treatment of facial melasma: the pigment trial. Journal of Drugs in Dermatology, 3(4), 377–381.	Paid. Not full access.
Balkrishnan, R., McMichael, A. J., Camacho, F. T., Saltzberg, F., Housman, T. S., Grummer, S., Feldman, S. R., & Chren, M. M. (2003). Development and validation of a health-related quality of life instrument for women with melasma. The British Journal of Dermatology, 149(3), 572–577. https://doi.org/10.1046/j.1365-2133.2003.05419.x	Lack of information on MASI or MELASQoL scores.
Balkrishnan, R., McMichael, A. J., Hu, J. Y., Camacho, F. T., Shew, K. R., Bouloc, A., Rapp, S. R., & Feldman, S. R. (2006). Correlates of health-related quality of life in women with severe facial blemishes. International Journal of Dermatology, 45(2), 111–115. https://doi.org/10.1111/j.1365-4632.2004.02371.x	Lack of information on MASI or MELASQoL scores.

Banchhor, S., Gupta, S., & Mahendra, A. (2024). Clinical and dermoscopic evaluation of periorbital melanosis and its psychological impact and effect on quality of life: A descriptive study. Cureus, 16(11), e74625. https://doi.org/10.7759/cureus.74625	Without MASI or MELASQoL
Bertold, C., Fontas, E., Singh, T., Gastaut, N., Ruitort, S., Wehrlen Pugliese, S., & Passeron, T. (2023). Efficacy and safety of a novel triple combination cream compared to Kligman's trio for melasma: A 24-week double-blind prospective randomized controlled trial. Journal of the European Academy of Dermatology and Venereology, 37(12), 2601–2607. https://doi.org/10.1111/jdv.19455	Missing baseline data.
Cassiano, D. P., Espósito, A. C. C., Hassun, K. M., Lima, E. V. A., Bagatin, E., & Miot, H. A. (2019). Early clinical and histological changes induced by microneedling in facial melasma: A pilot study. Indian Journal of Dermatology, Venereology and Leprology, 85(6), 638–641. https://doi.org/10.4103/ijdvl.IJDVL_44_19	Paid. Not full access.
Cassiano, D., Esposito, A. C. C., Hassun, K., Bagatin, E., Lima, M. M. D. A., Lima, E. V. A., Miot, L. D. B., & Miot, H. A. (2020). Efficacy and safety of microneedling and oral tranexamic acid in the treatment of facial melasma in women: An open, evaluator-blinded, randomized clinical trial. Journal of the American Academy of Dermatology, 83(4), 1176–1178. https://doi.org/10.1016/j.jaad.2020.02.002	Editorial Letter
Chalermchai, T., & Rummaneethorn, P. (2018). Effects of a fractional picosecond 1,064 nm laser for the treatment of dermal and mixed type melasma. Journal of Cosmetic and Laser Therapy, 20(3), 134–139. https://doi.org/10.1080/14764172.2017.1376098	Without MASI or MELASQoL
Cho, T.-H., Hong, S.-B., Ryou, J.-H., & Lee, M.-H. (2007). Quality of life in melasma. Korean Journal of Dermatology, 45, 232–236.	Full article not available.
Colpas, P. T., Miyashiro, C. A., Schalka, S., & Gomes Neto, A. (2019). Efficacy on reducing facial melasma pigmentation during summer time: A new alternative for the treatment of this condition. In Proceedings of the 24th World Congress of Dermatology, Milan, Italy.	Poster.
Colpas, P. T., Miyashiro, C. A., Schalka, S., & Gomes Neto, A. (2019). Safety and efficacy on reducing facial melasma pigmentation with an acid-free dermocosmetic. In Proceedings of the 24th World Congress of Dermatology, Milan, Italy.	Poster.
Costa, A., Moisés, T. A., Cordero, T., Alves, C. R., & Marmirori, J. (2010). Association of emblica, licorice and belides as an alternative to hydroquinone in the clinical treatment of melasma. Anais Brasileiros de Dermatologia, 85(5), 613–620. https://doi.org/10.1590/s0365-05962010000500003	Poster.
da Costa, A., Pereira, M., Moisés, T., Cordero, T., Silva, A. R., Amazonas, F. T. P., Bentivoglio, F., & Pereira, E. S. P. (2011). Evaluation of quality of life improvement in melasma patients, measured by the MELASQoL following the use of a botanical combination based on *Bellis*	Lack of information on MASI

perennis, *Glycyrrhiza glabra*, and *Phyllanthus emblica*. Surgical & Cosmetic Dermatology, 3(3), 207–212.	or MELASQoL scores.
Dabas, G., Vinay, K., Parsad, D., Kumar, A., & Kumaran, M. S. (2020). Psychological disturbances in patients with pigmentary disorders: a cross-sectional study. Journal of the European Academy of Dermatology and Venereology, 34(2), 392–399. https://doi.org/10.1111/jdv.15987	Without MASI or MELASQoL
Dayal, S., Sahu, P., & Dua, R. (2017). Combination of glycolic acid peel and topical 20% azelaic acid cream in melasma patients: efficacy and improvement in quality of life. Journal of Cosmetic Dermatology, 16(1), 35–42. https://doi.org/10.1111/jocd.12260	Paid. Not full access.
de Amorim, R. P., Barbosa, M. M. C., Cassiano, D. P., Esposito, A. C. C., Dias, M. O., de Abreu, A. F. T., Bagatin, E., & Miot, H. A. (2024). Sequential therapy with topical clobetasol for 14 days followed by hydroquinone versus hydroquinone alone in facial melasma treatment: a randomized, double-blind, controlled clinical trial. International Journal of Dermatology, 63(9), 1221–1226. https://doi.org/10.1111/ijd.17094	Missing baseline data.
De Belilovsky, C., Lachmann, N., & Chadoutaud, B. (2015). Specific quality of life questionnaires for the evaluation of cosmetic products: An essential tool. Journal of the American Academy of Dermatology, 72(5, Suppl. 1), AB62. https://doi.org/10.1016/j.jaad.2015.02.260	Poster.
de Moura, L. A. P., Pinto, J. M. N., & Teixeira, M. de S. (2016). Oral use of lingonberry (*Vaccinium vitis-idaea* L.) as an alternative for the treatment of melasma in adult women. Surgical & Cosmetic Dermatology, 8(4 Suppl. 1), S34–S39. https://doi.org/10.5935/scd1984-8773.2016677	Without MASI or MELASQoL
Dogramaci, A. C., Havlucu, D. Y., Inandi, T., & Balkrishnan, R. (2009). Validation of a melasma quality of life questionnaire for the Turkish language: the MelasQoL-TR study. The Journal of Dermatological Treatment, 20(2), 95–99. https://doi.org/10.1080/09546630802287553	Lack of information on MASI or MELASQoL scores.
Ejaz, A., Rao, S. E., Manzoor, A., & Niaz, A. (2016). Quality of life assessment in chronic skin disorders. Journal of Pakistan Association of Dermatologists, 25(2), 86–89.	Without MASI or MELASQoL
El-Husseiny, R., Rakha, N., & Sallam, M. (2020). Efficacy and safety of tranexamic acid 5% cream vs hydroquinone 4% cream in treating melasma: A split-face comparative clinical, histopathological, and antera 3D camera study. Dermatologic Therapy, 33(6), e14240. https://doi.org/10.1111/dth.14240	Biased experimental design (split-face)
El-Komy, M. H. M., Elnowaihy, S. W., Shamma, R. N., & Bedair, N. I. (2024). Efficacy and safety of a novel weekly topical metformin-loaded peel-off mask in the treatment of melasma: a split-face, placebo-controlled study. Clinical and Experimental Dermatology, 49(12), 1633–1640. https://doi.org/10.1093/ced/llae274	Full article not available.

Espósito, A., Cassiano, D., Hassum, K., Bagatin, E., Lima, E., Lima, M., Miot, L., & Miot, H. (2020). Efficacy and safety of microneedling and oral tranexamic acid in the treatment of facial melasma in women: An open, evaluator-blinded, randomized clinical trial. Journal of the American Academy of Dermatology, 83(4), 1006–1013. https://doi.org/10.1016/j.jaad.2020.02.002	Poster.
Fatma, F., Baati, I., Mseddi, M., Sallemi, R., Turki, H., & Masmoudi, J. (2016). The psychological impact of melasma: A report of 30 Tunisian women. Journal of the American Academy of Dermatology, 75(2), 385–392. https://doi.org/10.1016/j.jaad.2016.03.001	Full article not available.
Fleming, J., & Bashir, S. (2012). Combination (hydroquinone 5%, tretinoin 0.1%, and hydrocortisone 1%) cream in treating facial hyperpigmentation: A retrospective patient satisfaction study. Journal of the American Academy of Dermatology, 66(4, Suppl. 1), AB1. https://doi.org/10.1016/j.jaad.2011.11.012	Poster.
Goh, C. L., Chuah, S. Y., Tien, S., Thng, G., Vitale, M. A., & Delgado-Rubin, A. (2018). Double-blind, Placebo-controlled Trial to Evaluate the Effectiveness of Polypodium Leucotomos Extract in the Treatment of Melasma in Asian Skin: A Pilot Study. The Journal of Clinical and Aesthetic Dermatology, 11(3), 14–19.	Lack of information on MASI or MELASQoL scores.
Gold, M., DiBernardo, B., Rendon, M., & Bruce, S. (2012). Treatment of moderate to severe melasma in darker skin with a 4% hydroquinone skin care system plus 0.05% tretinoin cream. Journal of the American Academy of Dermatology, 66(4, Suppl. 1), AB180. https://doi.org/10.1016/j.jaad.2011.11.759	Poster.
Gold, M., Rendon, M., Dibernardo, B., Bruce, S., Lucas-Anthony, C., & Watson, J. (2013). Open-label treatment of moderate or marked melasma with a 4% hydroquinone skin care system plus 0.05% tretinoin cream. The Journal of Clinical and Aesthetic Dermatology, 6(11), 32–38.	Without MASI or MELASQoL
Grimes, P., & Watson, J. (2012). Treatment of melasma in darker skin with a 4% hydroquinone skin care system plus 0.025% tretinoin cream. Journal of the American Academy of Dermatology, 66(4, Suppl. 1), AB180. https://doi.org/10.1016/j.jaad.2011.11.758	Poster.
Gupta, S. (2019). To study the clinical profile and to assess the quality of life in 500 patients of melasma. In Proceedings of the 24th World Congress of Dermatology, Milan, Italy.	Poster.
Hammerschmidt, M., de Mattos, S. M. L., Suzuki, H. S., de Freitas, C. F. N. P., & Mukai, M. M. (2012). Evaluation of melasma classification methods based on response to treatment. Surgical & Cosmetic Dermatology, 4(2), 155–158.	Lack of information on MASI or MELASQoL scores.
Huang, P., Acevedo, S. F., Cheng, T., Mehta, R. C., & Makino, E. T. (2024). A randomized, controlled, split-face, double-blind comparison of a multimodality pigment-correcting serum containing lotus sprout extract versus hydroquinone for moderate to severe facial	Biased experimental design (split-face)

hyperpigmentation, including melasma, in a diverse population. JAAD International, 15, 206–219. https://doi.org/10.1016/j.jdin.2024.02.017	
Ikino, J. K., Nunes, D. H., Silva, V. P., Fröde, T. S., & Sens, M. M. (2015). Melasma and assessment of the quality of life in Brazilian women. Anais Brasileiros de Dermatologia, 90(2), 196–200. https://doi.org/10.1590/abd1806-4841.20152771	Without MASI or MELASQoL
Ismail, S. A., Mohamed, G. A., Mohamedeen, K. N., Sotohy, R. S. A., & Bakr, R. M. (2024). Does systemic metformin have a role in treating melasma? Dermatologic Surgery, 50(4), 366–371. https://doi.org/10.1097/DSS.0000000000004092	Paid. Not full access.
Kang, H. K., Baek, J. O., Roh, J. Y., & Lee, J. R. (2012). Change of quality of life after melasma treatment. Korean Journal of Dermatology, 50(7), 579–583.	Paid. Not full access.
Kang, H. K., Kim, J. H., Choi, J. S., Yun, J. H., Baek, J. O., Roh, J. Y., & Lee, J. R. (2012). Change of quality of life after melasma treatment. Journal of Dermatology, 39(Suppl. 1), 1–280. https://doi.org/10.1111/j.1346-8138.2012.01624.x	Poster.
Kaufman, B. P., & Alexis, A. F. (2020). Randomized, double-blinded, split-face study comparing the efficacy and tolerability of two topical products for melasma. Journal of Drugs in Dermatology, 19(9), 822–827. https://doi.org/10.36849/JDD.2020.10.36849/JDD.2020.5353	Paid. Not full access.
Keshavamurthy, V., Bhattacharjee, R., Hanumanthu, V., Thakur, V., Bishnoi, A., Parsad, D., & Kumaran, M. S. (2023). P70: A randomized open-label study to compare two different dosage regimens of oral tranexamic acid in treatment of moderate-to-severe facial melasma. British Journal of Dermatology, 188(Suppl. 4), ljad113.098. https://doi.org/10.1093/bjd/ljad113.098	Poster.
Kim, H.-Y., Park, G.-H., Park, E.-J., Kwon, I.-H., Kim, K.-H., & Kim, K.-J. (2013). Usefulness of melasma quality of life scale (MELASQOL) when evaluating the quality of life in Korean melasma patients. Korean Journal of Dermatology, 51, 422–428.	Full article not available.
Kim, W.-S. (2012). Effect and safety of the melasma peel (vitamin C peel plus oxygen) on skin pigmentary problem. Journal of Dermatology, 39(Suppl. 1), 1–280.	Poster.
Kirsch, B., Hoesly, P., & Sluzevich, J. (2018). Efficacy and tolerability of combination tazarotene, azelaic acid, tacrolimus, and zinc oxide for the treatment of melasma. Journal of the American Academy of Dermatology, 79(3, Suppl. 1), AB122.	Poster.
Lee, H. C., Thng, T. G. S., & Goh, C. L. (2016). Oral tranexamic acid (TA) in the treatment of melasma: A retrospective analysis. Journal of the American Academy of Dermatology, 75(2), 385–392. https://doi.org/10.1016/j.jaad.2016.03.019	Full article not available.

Lee, M. H., Noh, T. K., Lee, J. H., Roh, M. R., Na, J. I., Jung, E. C., Ko, J. Y., & Chang, S. E. (2016). Clinicoepidemiological features of melasma in Korean patients at five university hospitals: A cross-sectional multicenter study. Korean Journal of Dermatology, 54(7), 532–537.	Written in a non included language.
Leeyaphan, C., Wanitphakdeedecha, R., Manuskiatti, W., & Kulthanan, K. (2011). Measuring melasma patients' quality of life using willingness to pay and time trade-off methods in Thai population. BMC Dermatology, 11, 16. https://doi.org/10.1186/1471-5945-11-16	Without MASI or MELASQoL
Liu, H., Wang, J., Wang, X., & Zhang, F. (2012). Treatment of melasma with low fluence, 1064-nm Q-switched Nd:YAG laser in Chinese patients. Journal of Dermatology, 39(Suppl. 1), 1–280.	Poster.
Lyons, A. B., Moy, R. L., & Herrmann, J. L. (2019). A Randomized, controlled, split-face study of the efficacy of a picosecond laser in the treatment of melasma. Journal of Drugs in Dermatology, 18(11), 1104–1107.	Paid. Not full access.
Makino, E. T., Tan, P., & Mehta, R. C. (2017). Clinical efficacy and tolerability of a topical HQ-free serum on females with self-reported pregnancy-induced facial melasma. SKIN The Journal of Cutaneous Medicine, 1(3.1), 100. https://doi.org/10.25251/skin.1.supp.99	Poster.
Manzoni, A. P. D. S., Nogueira, J., Rizzatti, K., Lipnharski, C., Weber, M. B., & Lorenzini, F. K. (2019, June). Comparison between pulse-in-pulse intense pulsed light and 5% retinoic acid peeling for the treatment of melasma. In Proceedings of the 24th World Congress of Dermatology, Milan, Italy.	Poster.
Maranzatto, C. F. P., Miot, H. A., Miot, L. D. B., & Meneguin, S. (2016). Psychometric analysis and dimensional structure of the Brazilian version of melasma quality of life scale (MELASQoL-BP). Anais Brasileiros de Dermatologia, 91(4), 422–428. https://doi.org/10.1590/abd1806-4841.20165014	Duplicated data.
Martin, L. K., Caperton, C., Woolery-Lloyd, H., & Avashia, N. (2012). *A randomized double-blind placebo-controlled study evaluating the effectiveness and tolerability of oral Polypodium leucotomos in patients with melasma. Journal of the American Academy of Dermatology, 66*(4, Suppl. 1), AB21. https://doi.org/10.1016/j.jaad.2011.11.096	Poster.
Misery, L., Boussetta, S., Schmitt, A. M., Questel, E., & Taieb, C. (2009). Evaluation of the MELASQOL score of a population of French women. Acta Dermato-Venereologica, 89(5), 587. https://doi.org/10.2340/00015555-0726	Full article not available.
Misery, L., Schmitt, A. M., Boussetta, S., Rahhali, N., & Taieb, C. (2010). Melasma: measure of the impact on quality of life using the French version of MELASQOL after cross-cultural adaptation. *Acta Dermato-Venereologica*, *90*(3), 331–332. https://doi.org/10.2340/00015555-0837	Editorial Letter

Morita, Y., Kondo, T., Iijima, A., & Usuda, K. (2009). Covering foundation improves quality of life in patients with melasma. Skin Research, 8(4), 435–439.	Lack of information on MASI or MELASQoL scores.
Nagaraju, D., Bhattacharjee, R., Vinay, K., Saikia, U. N., Parsad, D., & Kumaran, M. S. (2018). Efficacy of oral tranexemic acid in refractory melasma: A clinico-immuno-histopathological study. Dermatologic Therapy, *31*(5), e12704. https://doi.org/10.1111/dth.12704	Lack of information on MASI or MELASQoL scores.
Pawaskar, M. D., Parikh, P., Markowski, T., McMichael, A. J., Feldman, S. R., & Balkrishnan, R. (2007). Melasma and its impact on health-related quality of life in Hispanic women. The Journal of Dermatological Treatment, 18(1), 5–9. https://doi.org/10.1080/09546630601028778	Lack of information on MASI or MELASQoL scores.
Pichardo, R., Vallejos, Q., Feldman, S. R., Schulz, M. R., Verma, A., Quandt, S. A., & Arcury, T. A. (2009). The prevalence of melasma and its association with quality of life in adult male Latino migrant workers. International Journal of Dermatology, 48(1), 22–26. https://doi.org/10.1111/j.1365-4632.2009.03778.x	Without MASI or MELASQoL
Platsidaki, E., Efstathiou, V., Markantoni, V., Kouris, A., Kontochristopoulos, G., Nikolaidou, E., Rigopoulos, D., Stratigos, A., & Gregoriou, S. (2023). Self-Esteem, Depression, Anxiety and Quality of Life in Patients with Melasma Living in a Sunny Mediterranean Area: Results from a Prospective Cross-Sectional Study. Dermatology and Therapy, 13(5), 1127–1136. https://doi.org/10.1007/s13555-023-00915-1	Lack of information on MASI or MELASQoL scores.
Pollo, C. F., Miot, L. D. B., Meneguin, S., & Miot, H. A. (2018). Factors associated with quality of life in facial melasma: a cross-sectional study. International Journal of Cosmetic Science, 10.1111/ics.12464. https://doi.org/10.1111/ics.12464	Lack of information on MASI or MELASQoL scores.
Pollo, C. F., Miot, L. D. B., Meneguin, S., & Miot, H. A. (2018). Development and validation of a multidimensional questionnaire for evaluating quality of life in melasma (HRQ-melasma). Anais Brasileiros de Dermatologia, 93(3), 391–396. https://doi.org/10.1590/abd1806-4841.20186780	Lack of information on MASI or MELASQoL scores.
Purim, K. S., & Avelar, M. F. (2012). Fotoproteção, melasma e qualidade de vida em gestantes [Photoprotection, melasma and quality of life in pregnant women]. Revista Brasileira de Ginecologia e Obstetricia, 34(5), 228–234. https://doi.org/10.1590/s0100-72032012000500007	Duplicated.
Qaiyyum, I. A., Nawab, M., & Kazmi, M. H. (2021). A randomized controlled clinical trial to evaluate safety and efficacy of a Unani formulation in the management of Kalaf (Melasma). Journal of Complementary & Integrative Medicine, 20(1), 233–240. https://doi.org/10.1515/jcim-2021-0353	Paid. Not full access.

Quandt, S. A., Schulz, M. R., Vallejos, Q. M., Feldman, S. R., Verma, A., Fleischer, A. B., Rapp, S. R., & Arcury, T. A. (2008). The association of dermatologist-diagnosed and self-reported skin diseases with skin-related quality of life in Latino migrant farmworkers. International Journal of Dermatology, 47(3), 236–241. https://doi.org/10.1111/j.1365-4632.2008.03518.x	Lack of information on MASI or MELASQoL scores.
Ramírez-Oliveros, J. F., de Abreu, L., Tamler, C., Vilhena, P., & de Barros, M. H. (2020). Microneedling with drug delivery (hydroquinone 4% serum) as an adjuvant therapy for recalcitrant melasma. Skinmed, 18(1), 38–40.	Full article not available.
Randomized, controlled, split-face study of combination therapy with topical methimazole plus low-power fractional CO2 laser vs. topical methimazole plus Q-switch laser for treatment of melasma. 2018. British Journal of Dermatology.	Poster.
Rendon M. I. (2004). Utilizing combination therapy to optimize melasma outcomes. Journal of Drugs in Dermatology, 3(5 Suppl), S27–S34.	Poster.
Rendon, M., & Dryer, L. (2016). Investigator-blinded, single-center study to evaluate the efficacy and tolerability of a 4% hydroquinone skin care system plus 0.02% tretinoin cream in mild-to-moderate melasma and photodamage. Journal of Drugs in Dermatology, 15(4), 466–475.	Paid. Not full access.
Rocha Mota, L., Motta, L. J., Duarte, I. D. S., Horliana, A. C. R. T., Silva, D. F. T. D., & Pavani, C. (2018). Efficacy of phototherapy to treat facial ageing when using a red versus an amber LED: a protocol for a randomised controlled trial. BMJ Open, 8(5), e021419. https://doi.org/10.1136/bmjopen-2017-021419	Study adapted the questionnaire.
Rodriguez-Arambula, A., Castanedo-Cazares, J. P., Hernandez-Blanco, D., & Torres-Alvarez, B. (2014). Melasma in Mexican women: A prevalence study of anxiety, depression, and its impact on quality of life. Journal of Investigative Dermatology, 134(Suppl. 1), S126–S126.	Poster.
Rosenthal, A., Mohamadi, K., & Moy, R. (2020). Addition of topical epidermal growth factor to microneedling treatments may enhance skin lightening benefits: A split-face pilot study for the management of melasma. Journal of the American Academy of Dermatology, 83(6, Suppl.), AB149. https://doi.org/10.1016/j.jaad.2020.06.683	Poster.
Rossi, A. B., Nocera, T., & Lapallud, P. (2016). Clinical efficacy of a dermocosmetic skin lightening cream in women suffering from melasma. Journal of the American Academy of Dermatology, 74(5, Suppl. 1), AB231. https://doi.org/10.1016/j.jaad.2016.02.912	Poster.
Seité, S., Deshayes, P., Dréno, B., Misery, L., Reygagne, P., Saiag, P., Stengel, F., Roguedas-Contios, A., & Rougier, A. (2012). Interest of corrective makeup in the management of patients in dermatology. Clinical, Cosmetic and Investigational Dermatology, 5, 123–128. https://doi.org/10.2147/CCID.S33172	Without MASI or MELASQoL

Sidorenko, O. A., Opruzhenkova, E. P., & Arkatova, E. A. (2022). Comparative study of the effectiveness of anti-aging cream with hydroquinone and broadband pulsed light in melasma. Russian Journal of Skin and Venereal Diseases, 25(3), 219–227. https://doi.org/10.17816/dv108987	Written in a non included language.
Taheri, R., Asadi, S., Ghorbani, N., & Ghorbani, R. (2016). Effects of Kligman-Willis's triple combination therapy on quality of life in female melasma patients. Koomesh Journal, 18(1), 220–226.	Written in a non included language.
Taylor, A., Pawaskar, M., Taylor, S. L., Balkrishnan, R., & Feldman, S. R. (2008). Prevalence of pigmentary disorders and their impact on quality of life: a prospective cohort study. Journal of Cosmetic Dermatology, 7(3), 164–168. https://doi.org/10.1111/j.1473-2165.2008.00384.x	Without MASI or MELASQoL
Tomar, N., Apekshaghai, Kaushik, M., Saleem, M., & Agarwal, M. (2020). Hyperpigmentation of skin (melasma) with solitary oral pyogenic granuloma lesion: A case report. Medico Legal Update, 20(4), 398–401. https://doi.org/10.37506/mlu.v20i4.1845	Case report.
Uyanikoglu, H., & Aksoy, M. (2017). Quality of life in patients with melasma in Turkish women. Dermatology Reports, 9(2), 7340. https://doi.org/10.4081/dr.2017.7340	Without MASI or MELASQoL
Wang, J. V., Christman, M. P., Feng, H., Ferzli, G., Jeon, H., & Geronemus, R. G. (2021). Laser-assisted delivery of tranexamic acid for melasma: Pilot study using a novel 1927 nm fractional thulium fiber laser. Journal of Cosmetic Dermatology, 20(1), 105–109. https://doi.org/10.1111/jocd.13817	Missing baseline data.
Wootton, C. I., Bell, S., Philavanh, A., Phommachack, K., Soukavong, M., Kidoikhammouan, S., Walker, S. L., & Mayxay, M. (2018). Assessing skin disease and associated health-related quality of life in a rural Lao community. BMC Dermatology, 18(1), 11. https://doi.org/10.1186/s12895-018-0079-8	Lack of information on MASI or MELASQoL scores.

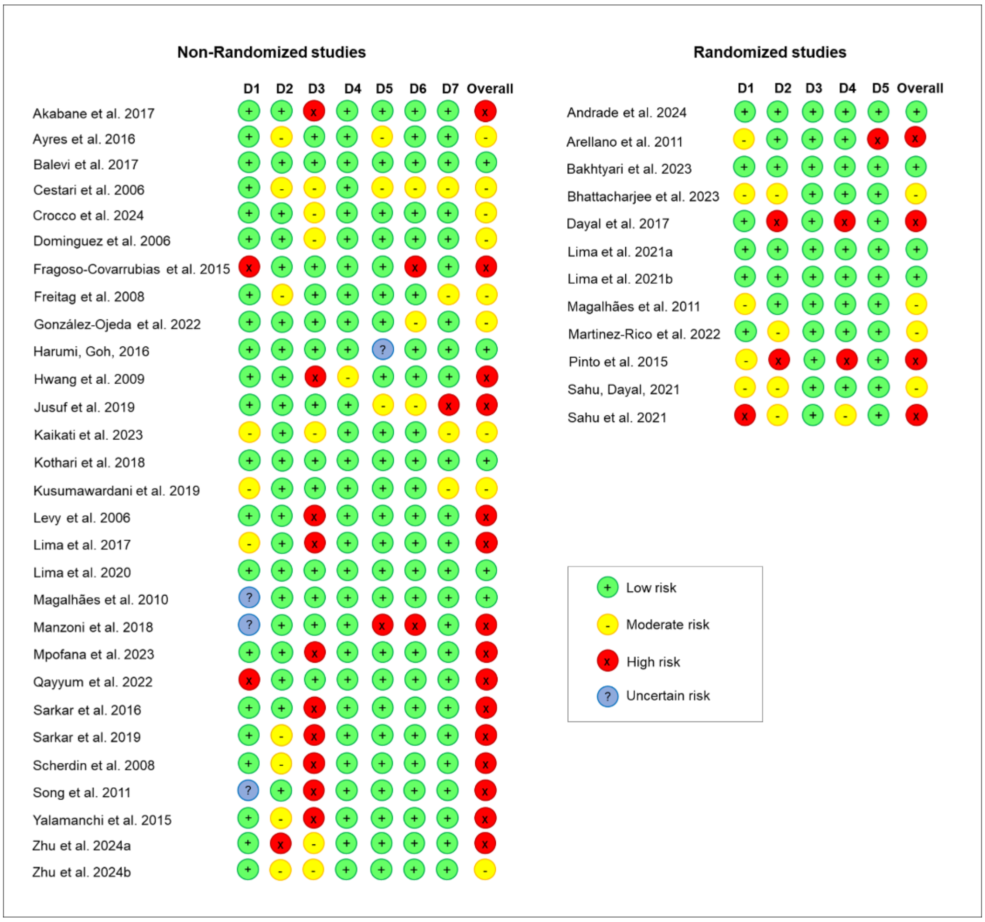

Figure S1. Quality analysis through risk of bias on non-randomized studies and randomized studies following the analysis of the respective domains: [D1]: bias due to confusing / randomization process [D2]: bias in the selection of participants into the study / deviations from the intended interventions; [D3]: bias in the measurement of interventions/ missing outcome data; [D4]: Bias due to departure from intended intervention/ measurement of the outcome; [D5]: Bias due to missing data / selection of the reported result; [D6]: bias in the measurement of outcomes; [D7]: Bias in selection of reported results.

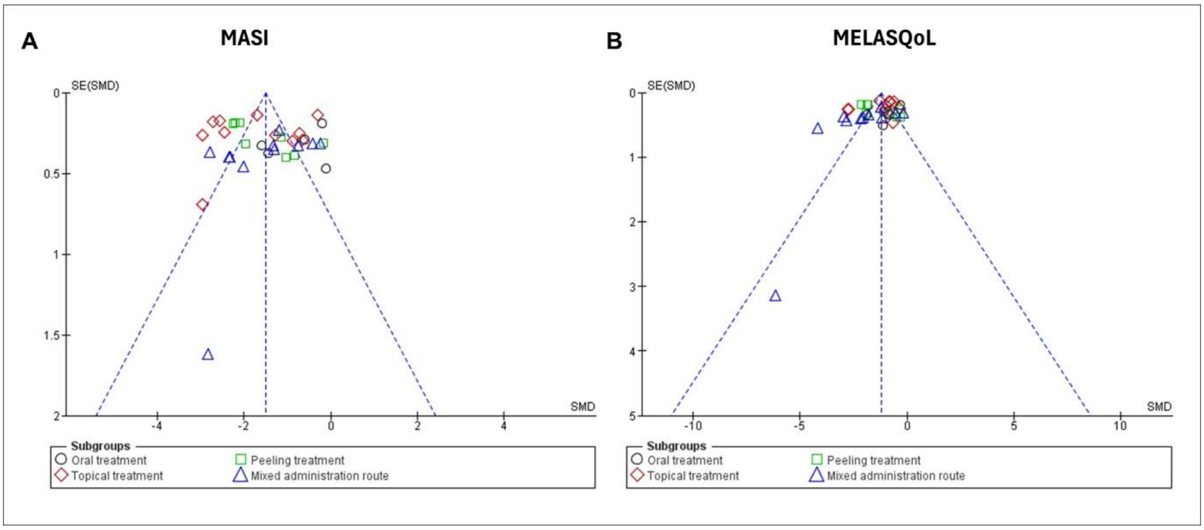

Figure S2. Funnel plot, respectively, from MASI and MELASQoL studies included in the meta-analysis.

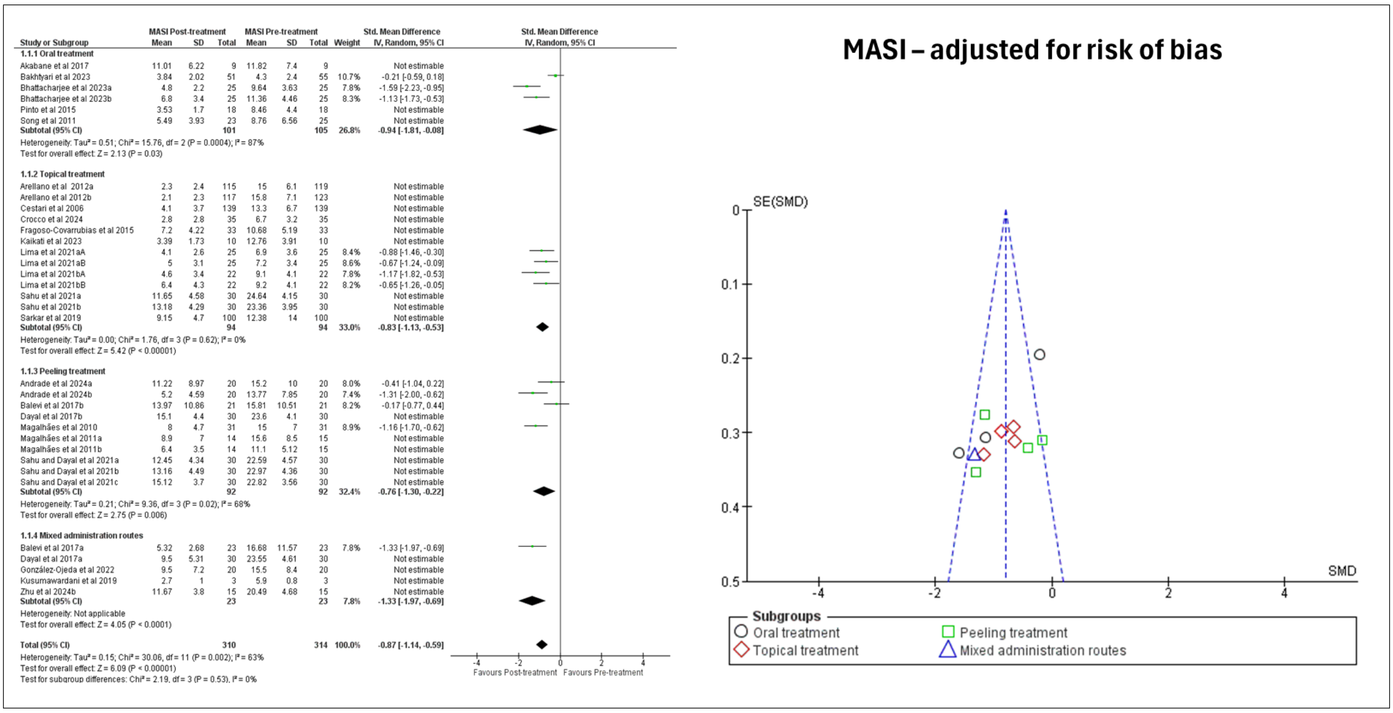

Figure S3. A sensitivity analysis was conducted to assess heterogeneity resulting from study bias. The MASI outcome from studies with a low risk of bias is presented in the forest and funnel plots.

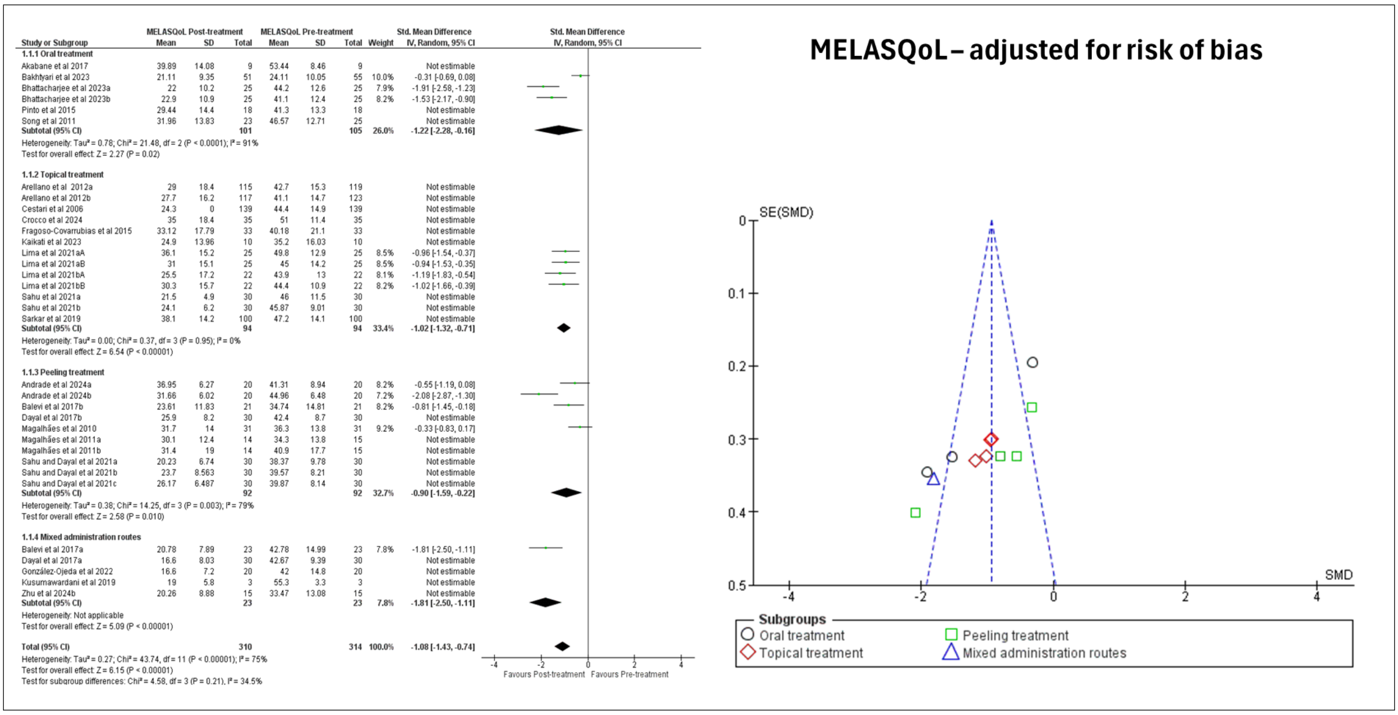

Figure S4. A sensitivity analysis was conducted to assess heterogeneity resulting from study bias. The MELASQoL outcome from studies with a low risk of bias is presented in the forest and funnel plots.

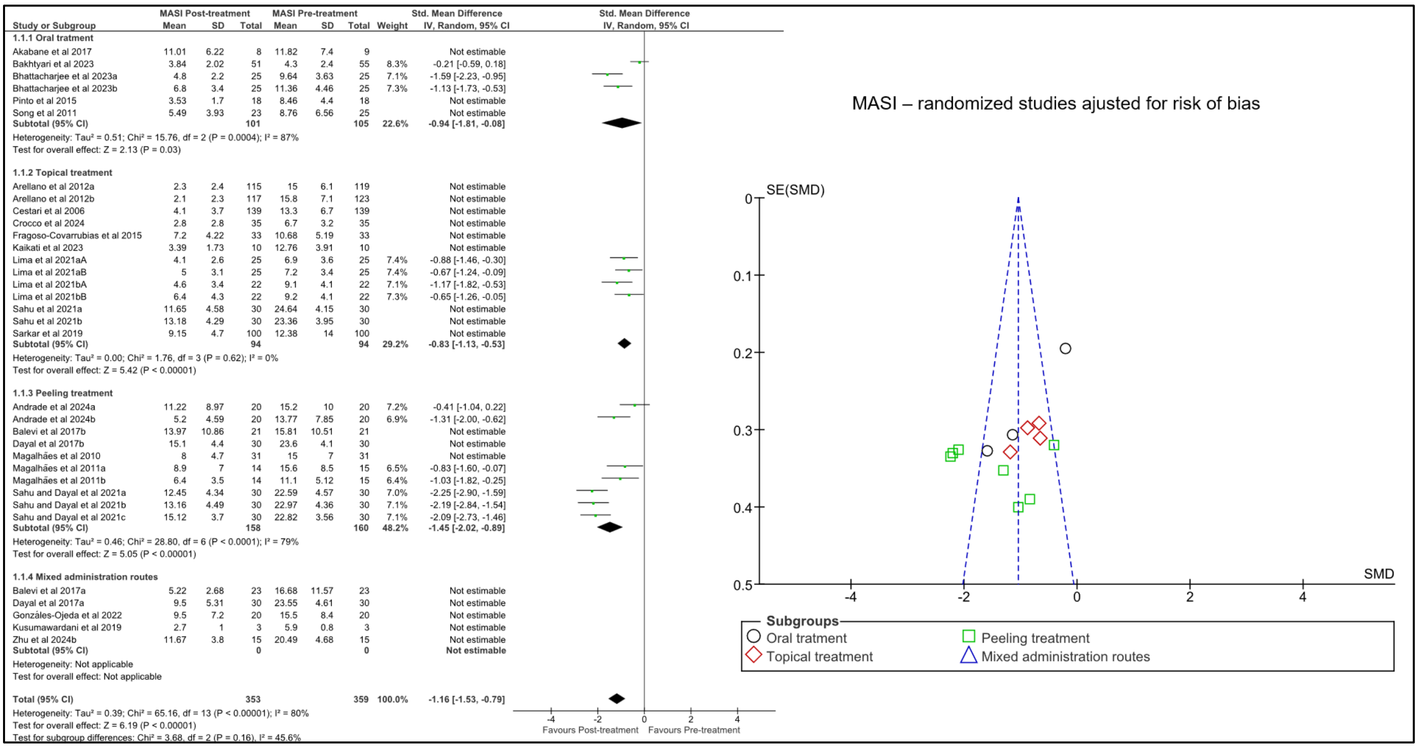

Figure S5. A sensitivity analysis was conducted to assess heterogeneity resulting from study design and study bias (moderate and low). The MASI outcome from randomized clinical trials with a moderate to low risk of bias is presented in the forest and funnel plots.

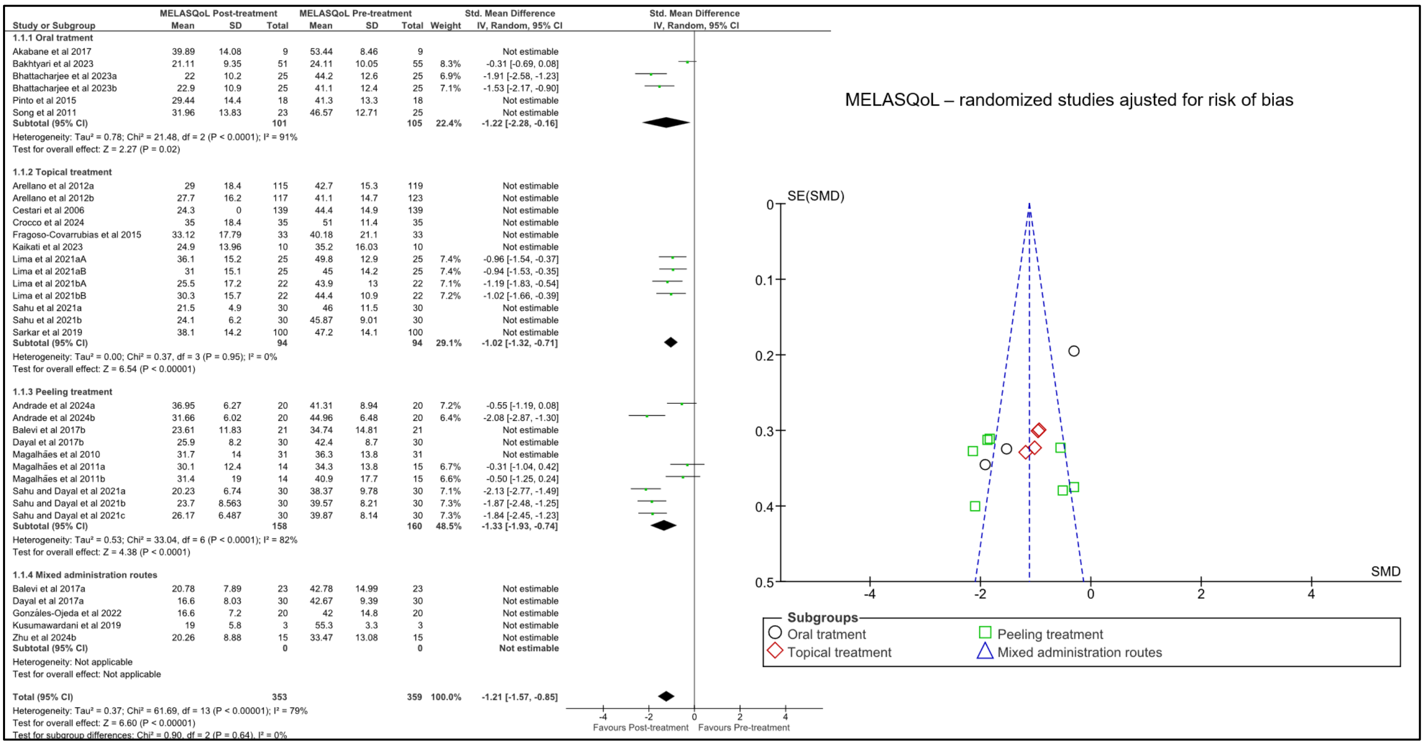

Figure S6. A sensitivity analysis was conducted to assess heterogeneity resulting from study design and study bias (moderate and low). The MELASQoL outcome from randomized clinical trials with a moderate to low risk of bias is presented in the forest and funnel plots.

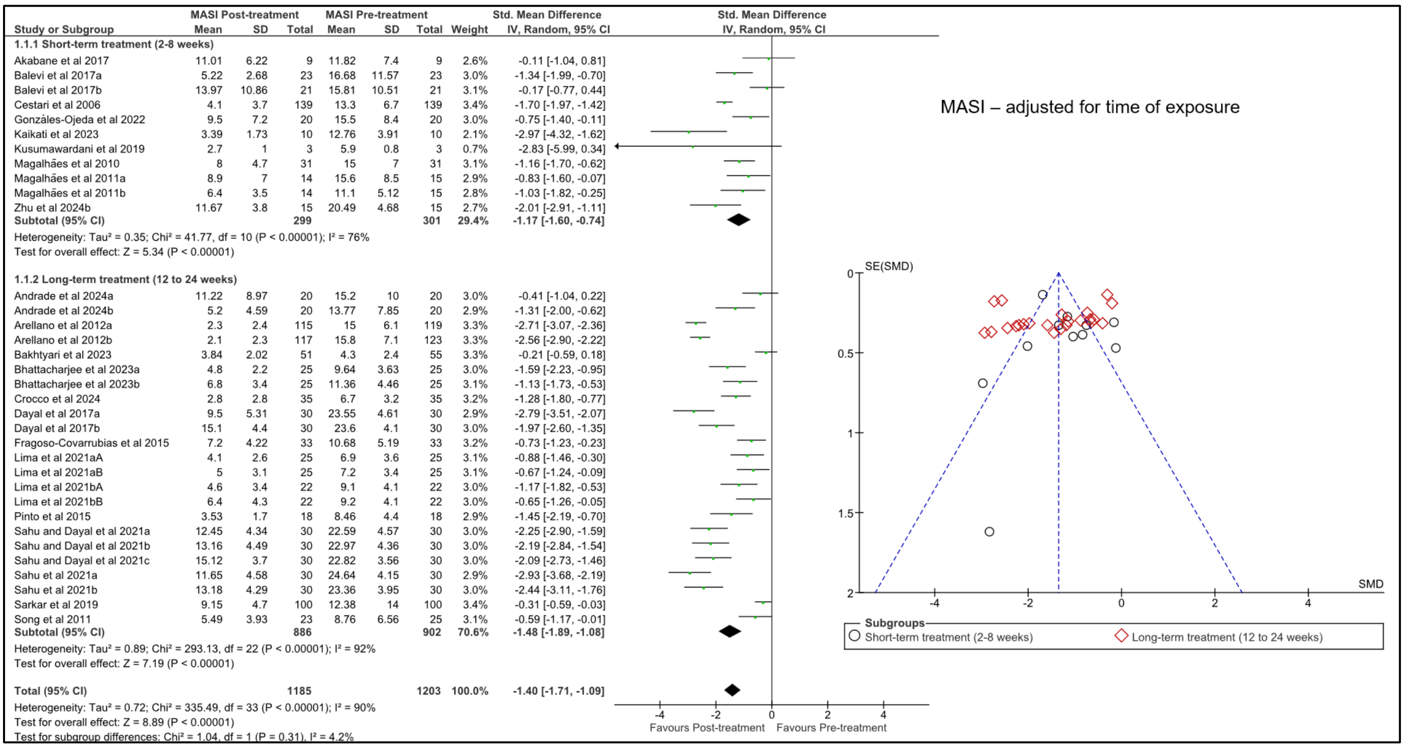

Figure S7. A sensitivity analysis was conducted to assess heterogeneity resulting from time of exposure. The MASI outcome from studies with short-term and long-term treatment is presented in the forest and funnel plots.

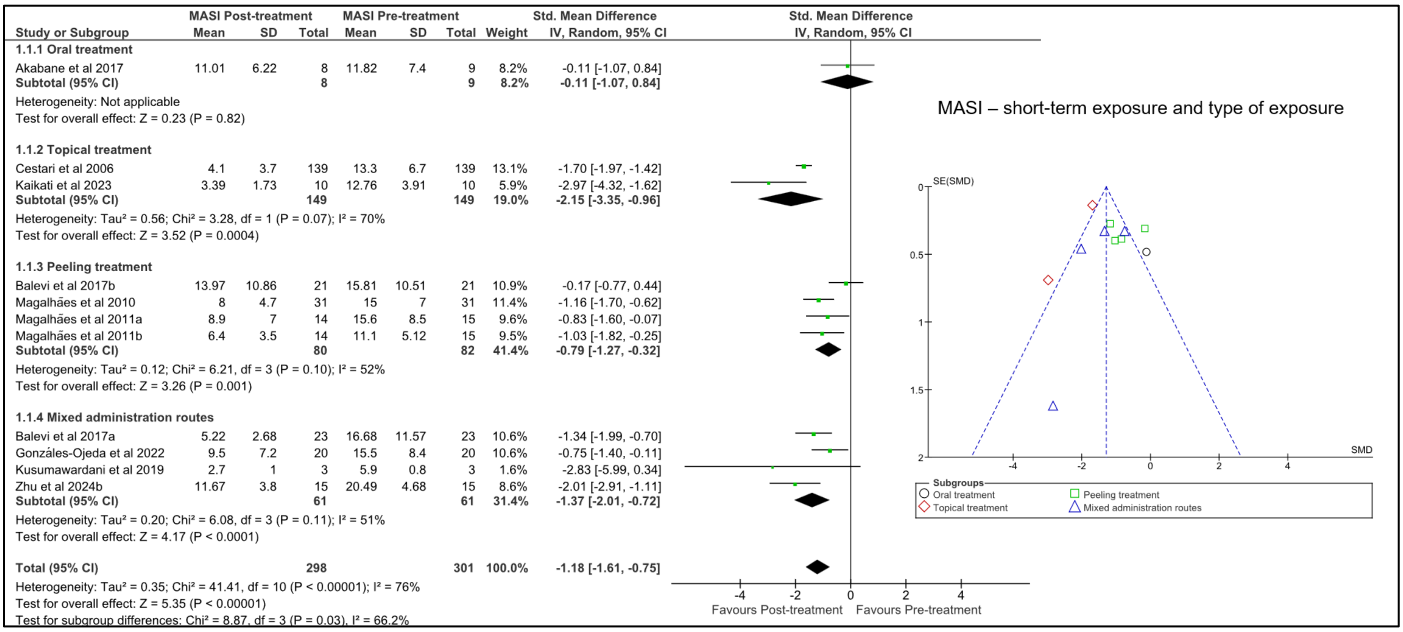

Figure S8. A sensitivity analysis was conducted to assess heterogeneity resulting from short-term exposure (2 to 8 weeks) and type of exposure. The MASI outcome from studies with short-term treatment is presented in the forest and funnel plots.

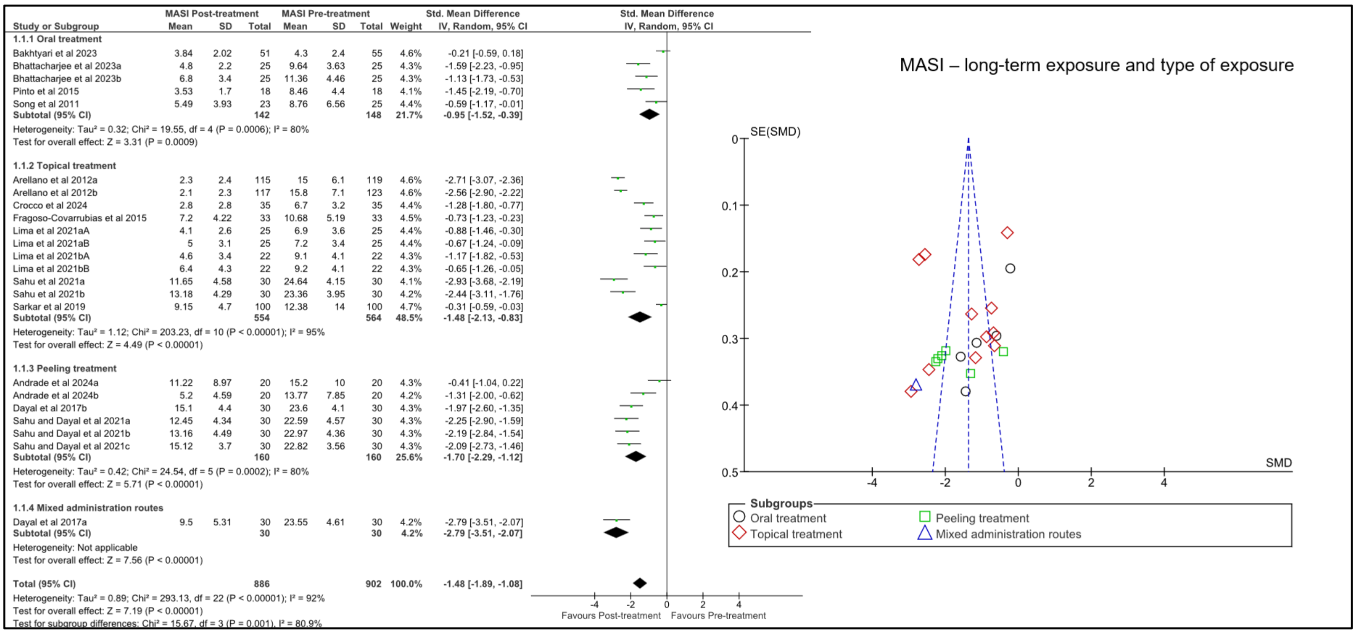

Figure S9. A sensitivity analysis was conducted to assess heterogeneity resulting from long-term exposure (12 to 24 weeks) and type of exposure. The MASI outcome from studies with long-term treatment is presented in the forest and funnel plots.

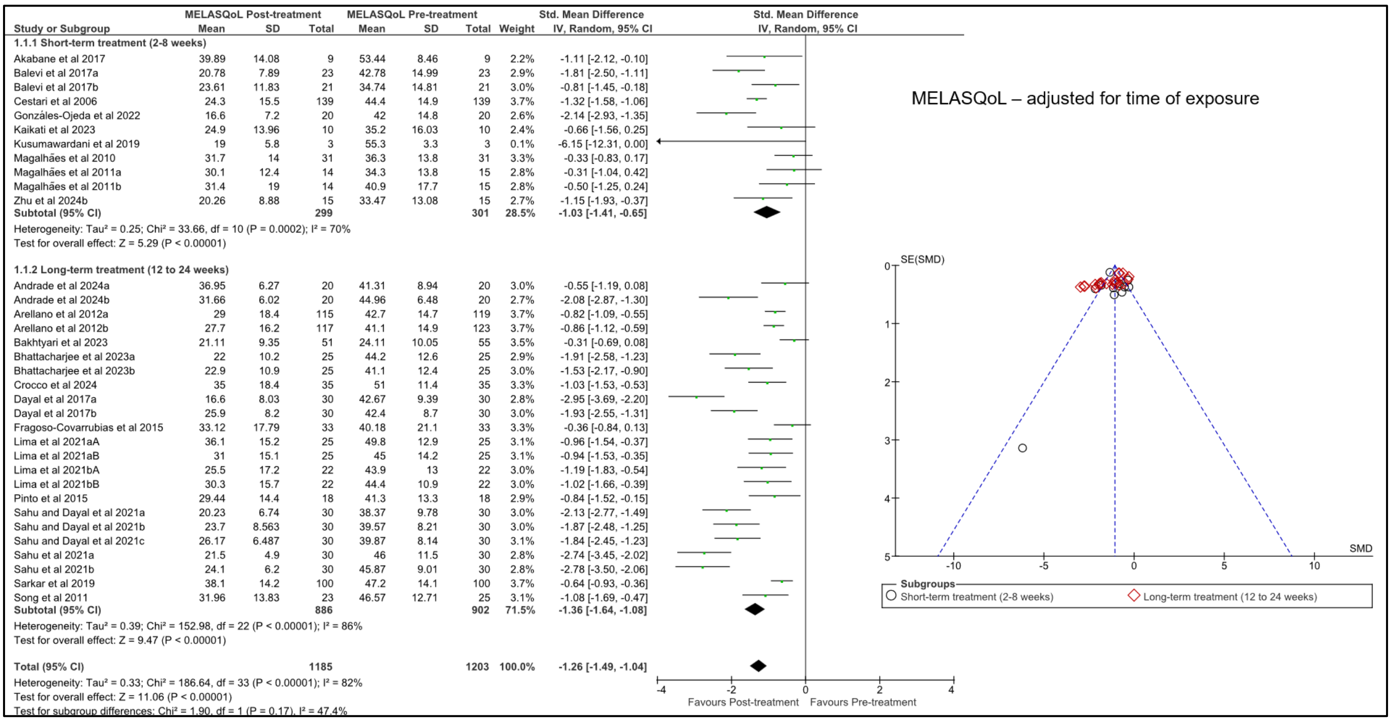

Figure S10. A sensitivity analysis was conducted to assess heterogeneity resulting from time of exposure. The MELASQoL outcome from studies with short-term and long-term treatment is presented in the forest and funnel plots.

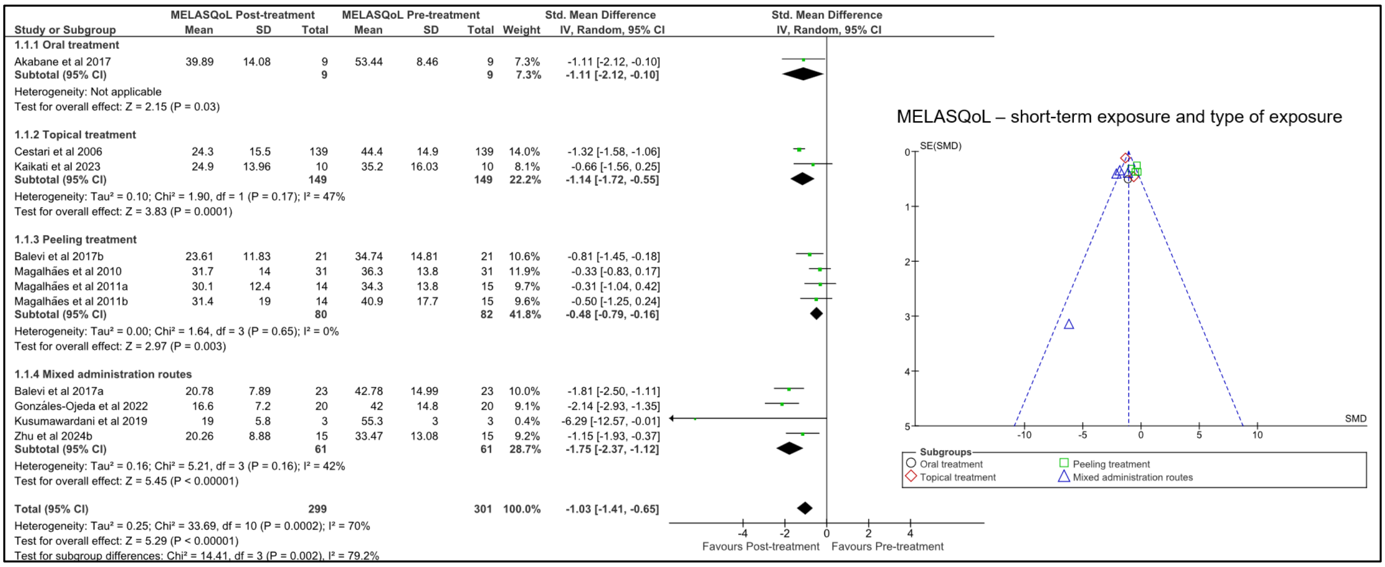

Figure S11. A sensitivity analysis was conducted to assess heterogeneity resulting from short-term exposure (2 to 8 weeks) and type of exposure. The MELASQoL outcome from studies with short-term treatment is presented in the forest and funnel plots.

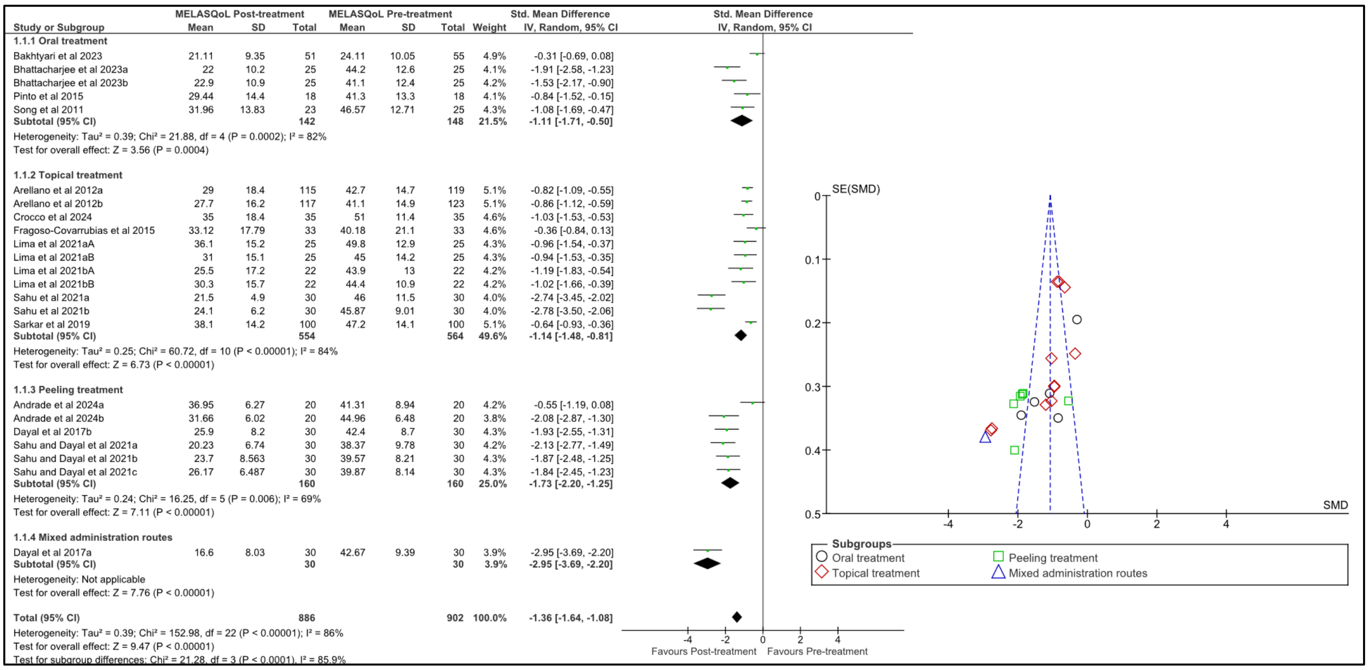

Figure S12. A sensitivity analysis was conducted to assess heterogeneity resulting from long-term exposure (12 to 24 weeks) and type of exposure. The MELASQoL outcome from studies with long-term treatment is presented in the forest and funnel plots.

Section and Topic	Item #	Checklist item	Reported (Yes/No)
**TITLE**			
Title	1	Identify the report as a systematic review.	Yes. Page 1
**BACKGROUND**			
Objectives	2	Provide an explicit statement of the main objective(s) or question(s) the review addresses.	Yes. Page 1
**METHODS**			
Eligibility criteria	3	Specify the inclusion and exclusion criteria for the review.	Yes. Page 1
Information sources	4	Specify the information sources (e.g. databases, registers) used to identify studies and the date when each was last searched.	Yes. Page 1
Risk of bias	5	Specify the methods used to assess risk of bias in the included studies.	No.
Synthesis of results	6	Specify the methods used to present and synthesise results.	No.
**RESULTS**			
Included studies	7	Give the total number of included studies and participants and summarise relevant characteristics of studies.	Yes. Page 1.
Synthesis of results	8	Present results for main outcomes, preferably indicating the number of included studies and participants for each. If meta-analysis was done, report the summary estimate and confidence/credible interval. If comparing groups, indicate the direction of the effect (i.e. which group is favoured).	Yes. Page 1.
**DISCUSSION**			
Limitations of evidence	9	Provide a brief summary of the limitations of the evidence included in the review (e.g. study risk of bias, inconsistency and imprecision).	No.
Interpretation	10	Provide a general interpretation of the results and important implications.	Yes. Page 1.
**OTHER**			
Funding	11	Specify the primary source of funding for the review.	Out of the scope.
Registration	12	Provide the register name and registration number.	Out of the scope.

Figure S13. PRISMA 2020 for abstracts checklist.

Section and Topic	Item #	Checklist item	Location where item is reported
**TITLE**			
Title	1	Identify the report as a systematic review.	Page 1.
**ABSTRACT**			
Abstract	2	See the PRISMA 2020 for Abstracts checklist.	Fig. S13.
**INTRODUCTION**			
Rationale	3	Describe the rationale for the review in the context of existing knowledge.	Page 1 – 3.
Objectives	4	Provide an explicit statement of the objective(s) or question(s) the review addresses.	Page 3.
**METHODS**			
Eligibility criteria	5	Specify the inclusion and exclusion criteria for the review and how studies were grouped for the syntheses.	Page 21. (4.1.2. Eligibility criteria)
Information sources	6	Specify all databases, registers, websites, organisations, reference lists and other sources searched or consulted to identify studies. Specify the date when each source was last searched or consulted.	Page 21. (4.1.1. Search Strategy)
Search strategy	7	Present the full search strategies for all databases, registers and websites, including any filters and limits used.	Page 21. (4.1.1. Search Strategy)
Selection process	8	Specify the methods used to decide whether a study met the inclusion criteria of the review, including how many reviewers screened each record and each report retrieved, whether they worked independently, and if applicable, details of automation tools used in the process.	Page 21. (4.1.3. Data extraction)
Data collection process	9	Specify the methods used to collect data from reports, including how many reviewers collected data from each report, whether they worked independently, any processes for obtaining or confirming data from study investigators, and if applicable, details of automation tools used in the process.	Page 21. (4.1.3. Data extraction)
Data items	10a	List and define all outcomes for which data were sought. Specify whether all results that were compatible with each outcome domain in each study were sought (e.g. for all measures, time points, analyses), and if not, the methods used to decide which results to collect.	Page 21. (4.1.3. Data extraction)
	10b	List and define all other variables for which data were sought (e.g. participant and intervention characteristics, funding sources). Describe any assumptions made about any missing or unclear information.	Not included.
Study risk of bias assessment	11	Specify the methods used to assess risk of bias in the included studies, including details of the tool(s) used, how many reviewers assessed each study and whether they worked independently, and if applicable, details of automation tools used in the process.	Page 21. (4.2 Quality assessment)
Effect	12	Specify for each outcome the effect measure(s) (e.g. risk ratio, mean difference) used in the	Page 22.

Section and Topic	Item #	Checklist item	Location where item is reported
measures		synthesis or presentation of results.	(4.3 Statistical analysis)
Synthesis methods	13a	Describe the processes used to decide which studies were eligible for each synthesis (e.g. tabulating the study intervention characteristics and comparing against the planned groups for each synthesis (item #5)).	Out of the scope.
	13b	Describe any methods required to prepare the data for presentation or synthesis, such as handling of missing summary statistics, or data conversions.	Out of the scope.
	13c	Describe any methods used to tabulate or visually display results of individual studies and syntheses.	Page 22. (4.3 Statistical analysis)
	13d	Describe any methods used to synthesize results and provide a rationale for the choice(s). If meta-analysis was performed, describe the model(s), method(s) to identify the presence and extent of statistical heterogeneity, and software package(s) used.	Page 22. (4.3 Statistical analysis)
	13e	Describe any methods used to explore possible causes of heterogeneity among study results (e.g. subgroup analysis, meta-regression).	Page 22. (4.3 Statistical analysis)
	13f	Describe any sensitivity analyses conducted to assess robustness of the synthesized results.	Page 22. (4.3 Statistical analysis)
Reporting bias assessment	14	Describe any methods used to assess risk of bias due to missing results in a synthesis (arising from reporting biases).	Page 22. (4.3 Statistical analysis)
Certainty assessment	15	Describe any methods used to assess certainty (or confidence) in the body of evidence for an outcome.	Out of the scope.
**RESULTS**			
Study selection	16a	Describe the results of the search and selection process, from the number of records identified in the search to the number of studies included in the review, ideally using a flow diagram.	Page 3 and Fig. 1.
	16b	Cite studies that might appear to meet the inclusion criteria, but which were excluded, and explain why they were excluded.	Table S1.
Study characteristics	17	Cite each included study and present its characteristics.	Table 1 and 2.
Risk of bias in studies	18	Present assessments of risk of bias for each included study.	Fig. S1
Results of individual studies	19	For all outcomes, present, for each study: (a) summary statistics for each group (where appropriate) and (b) an effect estimate and its precision (e.g. confidence/credible interval), ideally using structured tables or plots.	Table 1 and 2.

Section and Topic	Item #	Checklist item	Location where item is reported
Results of syntheses	20a	For each synthesis, briefly summarise the characteristics and risk of bias among contributing studies.	Page 4. (2.1. Quality assessment)
	20b	Present results of all statistical syntheses conducted. If meta-analysis was done, present for each the summary estimate and its precision (e.g. confidence/credible interval) and measures of statistical heterogeneity. If comparing groups, describe the direction of the effect.	Pages 5 and 14-15.
	20c	Present results of all investigations of possible causes of heterogeneity among study results.	Pages 5 and 14-15.
	20d	Present results of all sensitivity analyses conducted to assess the robustness of the synthesized results.	Pages 5 and 14-15.
Reporting biases	21	Present assessments of risk of bias due to missing results (arising from reporting biases) for each synthesis assessed.	Out of scope.
Certainty of evidence	22	Present assessments of certainty (or confidence) in the body of evidence for each outcome assessed.	Out of the scope.
**DISCUSSION**			
Discussion	23a	Provide a general interpretation of the results in the context of other evidence.	Page 18-21.
	23b	Discuss any limitations of the evidence included in the review.	Page 18-21.
	23c	Discuss any limitations of the review processes used.	Page 18-21.
	23d	Discuss implications of the results for practice, policy, and future research.	Page 18-21.
**OTHER INFORMATION**			
Registration and protocol	24a	Provide registration information for the review, including register name and registration number, or state that the review was not registered.	Page 21. (4.1 Systematic review)
	24b	Indicate where the review protocol can be accessed, or state that a protocol was not prepared.	Page 21. (4.1 Systematic review)
	24c	Describe and explain any amendments to information provided at registration or in the protocol.	Out of the scope.
Support	25	Describe sources of financial or non-financial support for the review, and the role of the funders or sponsors in the review.	Page 23.
Competing interests	26	Declare any competing interests of review authors.	Page 23.
Availability of data, code and other	27	Report which of the following are publicly available and where they can be found: template data collection forms; data extracted from included studies; data used for all analyses; analytic code; any other materials used in the review.	Page 23.

Section and Topic	Item #	Checklist item	Location where item is reported
materials			

Figure S14. PRISMA 2020 checklist.
